# Interploidy Introgression Shaped Adaptation during the Origin and Domestication History of *Brassica napus*

**DOI:** 10.1093/molbev/msad199

**Published:** 2023-09-14

**Authors:** Tianpeng Wang, Aalt D J van Dijk, Johan Bucher, Jianli Liang, Jian Wu, Guusje Bonnema, Xiaowu Wang

**Affiliations:** State Key Laboratory of Vegetable Biobreeding, Institute of Vegetables and Flowers, Chinese Academy of Agricultural Sciences, Beijing, China; Sino-Dutch Joint Laboratory of Horticultural Genomics, Institute of Vegetables and Flowers, Chinese Academy of Agricultural Sciences, Beijing, China; Plant Breeding, Wageningen University and Research, Wageningen, The Netherlands; Bioinformatics Group, Wageningen University and Research, Wageningen, The Netherlands; Bioinformatics Group, Wageningen University and Research, Wageningen, The Netherlands; Plant Breeding, Wageningen University and Research, Wageningen, The Netherlands; State Key Laboratory of Vegetable Biobreeding, Institute of Vegetables and Flowers, Chinese Academy of Agricultural Sciences, Beijing, China; Sino-Dutch Joint Laboratory of Horticultural Genomics, Institute of Vegetables and Flowers, Chinese Academy of Agricultural Sciences, Beijing, China; State Key Laboratory of Vegetable Biobreeding, Institute of Vegetables and Flowers, Chinese Academy of Agricultural Sciences, Beijing, China; Sino-Dutch Joint Laboratory of Horticultural Genomics, Institute of Vegetables and Flowers, Chinese Academy of Agricultural Sciences, Beijing, China; Sino-Dutch Joint Laboratory of Horticultural Genomics, Institute of Vegetables and Flowers, Chinese Academy of Agricultural Sciences, Beijing, China; Plant Breeding, Wageningen University and Research, Wageningen, The Netherlands; State Key Laboratory of Vegetable Biobreeding, Institute of Vegetables and Flowers, Chinese Academy of Agricultural Sciences, Beijing, China; Sino-Dutch Joint Laboratory of Horticultural Genomics, Institute of Vegetables and Flowers, Chinese Academy of Agricultural Sciences, Beijing, China

**Keywords:** Brassica, polyploidy, interploidy introgression, adaptive introgression, origin, domestication, *Brassica napus*

## Abstract

Polyploidy is recurrent across the tree of life and known as an evolutionary driving force in plant diversification and crop domestication. How polyploid plants adapt to various habitats has been a fundamental question that remained largely unanswered. *Brassica napus* is a major crop cultivated worldwide, resulting from allopolyploidy between unknown accessions of diploid *B. rapa* and *B. oleracea*. Here, we used whole-genome resequencing data of accessions representing the majority of morphotypes and ecotypes from the species *B. rapa*, *B. oleracea*, and *B. napus* to investigate the role of polyploidy during domestication. To do so, we first reconstructed the phylogenetic history of *B. napus*, which supported the hypothesis that the emergence of *B. napus* derived from the hybridization of European turnip of *B. rapa* and wild *B. oleracea*. These analyses also showed that morphotypes of swede and Siberian kale (used as vegetable and fodder) were domesticated before rapeseed (oil crop). We next observed that frequent interploidy introgressions from sympatric diploids were prominent throughout the domestication history of *B. napus*. Introgressed genomic regions were shown to increase the overall genetic diversity and tend to be localized in regions of high recombination. We detected numerous candidate adaptive introgressed regions and found evidence that some of the genes in these regions contributed to phenotypic diversification and adaptation of different morphotypes. Overall, our results shed light on the origin and domestication of *B. napus* and demonstrate interploidy introgression as an important mechanism that fuels rapid diversification in polyploid species.

## Introduction

Polyploidy, referring to the condition in which cells or organisms possess more than two complete sets of chromosomes, has long been recognized as an important feature in plant history (Stebbins 1971; Hilu 1993; Soltis, et al. 2009). Extensive research over the recent decades has shown that polyploidy is far more prevalent than previously thought in the evolutionary history of plants, with many plant lineages having experienced several rounds of whole-genome duplications over time (Landis, et al. 2018; Leebens-Mack, et al. 2019; Zhao, et al. 2021). This widespread and recurrent status of polyploidy has been considered one of the main driving forces to phenotypic diversification and genome evolution in plants (Soltis, et al. 2015; Van de Peer, et al. 2017, 2020). Intriguingly, many domesticated crops have been demonstrated to be nascent polyploids, and some of which have even undergone multiple rounds of polyploidy events, suggesting that polyploidy can confer preconditions for successful domestication (Udall and Wendel 2006; Cheng, et al. 2014; Renny-Byfield and Wendel 2014; Salman-Minkov, et al. 2016). However, following polyploidization, multiple changes that lead to genomic instabilities can arise, especially when coupled with hybridization as is the case in allopolyploid species. These can include meiotic instability, perturbed gene expression, and epigenetic shock, which can have a direct negative influence on survival and adaptation, depicted as “evolutionary dead end” (Arrigo and Barker 2012; Soltis, et al. 2014; Nieto Feliner, et al. 2020). Understanding the genetic mechanisms of polyploidy that leads to successful domestication is of fundamental importance for a full appreciation of the potential of crops.

There is growing evidence that introgressive hybridization (introgression) across a ploidy barrier can take place at the early stages of polyploid adaptation (Chapman and Abbott 2010; Marburger, et al. 2019; Schmickl and Yant 2021). Introgressive hybridization between species is a well-documented process across the tree of life, which can result in the transfer of small amounts of genetic material from one species into another following recurrent backcrossing (Rieseberg and Carney 1998; Baack and Rieseberg 2007). The establishment of a new polyploid species requires a degree of reproductive isolation to remain distinct from its parental species (De Queiroz 2007). Stebbins (1971) has emphasized the potential contributions of introgressions in the early stages of polyploid evolution and pointed out that introgression mainly occurred unidirectionally, from a diploid to a tetraploid species (Stebbins 1971). Such unidirectional introgression has been documented in a handful of studies that show the morphological similarity of tetraploid species to their local diploid species (Kim, et al. 2008; Chapman and Abbott 2010; Whitney, et al. 2010; Han, et al. 2015; Zohren, et al. 2016). Interploidy gene flow can provide a source of novelty for transferring genetic variations into nascent polyploids, thereby permitting polyploids to better adapt to new ecological niches (Schmickl and Yant 2021; Liu, et al. 2022).

Interploidy gene flow is not only relevant during the initial establishment of polyploid species. Even when polyploid species have differentiated to such an extent that they adapt to disparate distribution regions, hybridization might occur after secondary contact, which is especially common postdomestication in crop species (Dempewolf, et al. 2017; Janzen, et al. 2019). Crop domestication is an evolutionary process arising from ancestral species within source centers, followed by expansion to the current distribution ranges (Meyer, et al. 2012). The role of hybridization in crop diversification has been widely reported, and there is evidence for gene flow from wild relatives in domestication, for polyploid crops such as wheat (He, et al. 2019; Zhou, et al. 2020), banana (Cenci, et al. 2020), maize (Wang, et al. 2017), and Brassica (Zhang, et al. 2022). This interploidy gene flow can lead to adaptive introgression, enabling the domesticated polyploid species to be used in new agricultural environments or adapt to new cultural preferences (Schmickl and Yant 2021). Still, despite the prevalence of cross-ploidy introgression in polyploid crop domestication, it is currently not clear to what extent interploidy gene flow affects species morphology and ecology.

The *Brassica* genus is a relevant target system for explorations of the effects of interploidy gene flow on adaptation and domestication, as it includes both three diploid and three allopolyploid species, resulting from their pairwise hybridization (Nagaharu 1935; Cheng, et al. 2017). Together the relationship of those six species is depicted as the “triangle of U.” In addition, the *Brassica* genus comprises highly diverse morphotypes within species as a result of artificial selection during domestication in different regions of the world (Cheng, et al. 2014, 2017). Among the *Brassica* species, *Brassica napus* (AC genome) provides a well-established study system because of its worldwide cultivation and economic importance and some favorable attributes, including the availability of well-established genomes, genetic transformation, and ease of resynthesis (Heslop-Harrison 2013; Chalhoub, et al. 2014; Song, et al. 2020). *Brassica napus* is an allopolyploid species, which has diversified into three recognized subspecies, including oil-type *B. napus* subsp. *oleifera* (rapeseed or oilseeds), tuber-type *B. napus* subsp. *rapifera* (swede or rutabaga), and leafy-type *B. napus* subsp*. pabularia* (Siberian kale or leaf rape) (Chalhoub, et al. 2014; Havlickova, et al. 2018). These morphotypes can be further clustered according to their growth habitats into winter ecotypes that require vernalization, semiwinter ecotypes that need a mild winter environment, and spring ecotypes that do not need cold treatment (Leijten, et al. 2018).

Understanding the phylogenetic relationship of a crop species is the fundamental first step in resolving subsequent analyses related to its domestication history. As one of the earlier allopolyploid crops, *B. napus* originated from hybridization of *B. rapa* (A genome) and *B. oleracea* (C genome) followed by polyploidization around 7500–12,500 years ago (Chalhoub, et al. 2014). Recent studies based on large whole-genome resequencing data of oil-type *B. napus* accessions have suggested that its direct A progenitor is the European turnip (*B. rapa*) and its C subgenome may have derived from the common ancestor of kohlrabi, cauliflower, broccoli, and Chinese kale (Yang, et al. 2016; An, et al. 2019; Lu, et al. 2019; Wu, et al. 2019). Still, its phylogenetic relationship and domestication history remain elusive due to insufficient sampling of wild *B. oleracea* accessions, and the leafy and swede subspecies of *B. napus*. *Brassica napus* is presumed to have originated geographically in the European-Central Asian regions, where ancient morphotypes of *B. rapa* and *B. oleracea* coexisted (Qi, et al. 2017; Mabry, et al. 2021). During the modern domestication process, different *B. napus* morphotypes have largely been cultivated sympatrically with their diploid progenitor species. Intraspecies introgression has been reported among populations of different wild and diversified crops of diploid species (McAlvay, et al. 2021; Cai, et al. 2022; Saban, et al. 2023). Interploidy introgression can happen through triploid bridges and can result in transfer of desired traits from diploids into allopolyploids resulting in genotypes with improved adaptive value (Mason and Batley 2015). Previous studies have revealed some potential interploidy introgression events in *B. napus* (Sun, et al. 2017; Zou, et al. 2019). In addition, active intercrossing between *B. rapa* and *B. napus* rapeseed were performed in China and Australia to increase *B. napus* genetic diversity (Udall, et al. 2004; Qian, et al. 2005; Chen, Zou, et al. 2010). This breeding approach has been successful in developing new rapeseed varieties with desirable traits such as high yield, disease resistance, and improved oil quality (Chatterjee, et al. 2016; Mei, et al. 2020; Zhang, et al. 2022). However, the overall extent and genomic location of introgressed regions remain unexplored among different *B. napus* morphotypes and so do the potential functional and adaptive values that may influence successful domestication.

In this study, by generating whole-genome resequencing data together with public data representing major morphotypes of *B. rapa*, *B. oleracea*, and *B. napus*, we analyzed the origin and demographic history of *B. napus*. We then perform a cross-ploidy comparison of genomic context between different morphotypes and examine the occurrence of interploidy introgression during *B. napus* domestication. Finally, we qualified the genomic patterns of introgressed regions and clarified how potential interploidy introgression shaped the functional adaptation among the specific morphotypes during their domestication.

## Results

### Sequencing and Cross-Ploidy Variations Discovery

To fully present genetic variation between and within the morphotypes in *B. napus* and its progenitors *B. rapa* and *B. oleracea*, we collected publicly available resequencing data of large collections of these three species and generated resequencing data of 33 additional accessions of morphotypes with lower representation in these collections. This resulted in a total of 614 accessions from 4 species (*B. napus*, 283 accessions; *B. rapa,* 199 accessions; *B. oleracea,* 130 accessions; and *B. nigra*, 2 accessions), representing a wide range of ploidy levels, subspecies, and geographic distribution (supplementary table S1, Supplementary Material online). For *B. napus*, our dataset represents all the morphotypes and ecotypes, including swede, Siberian kale, winter rapeseed, spring rapeseed, and semi-winter rapeseed. Given the different ploidy levels between *B. rapa* (AA), *B. oleracea* (CC), and *B. napus* (AACC), the resequencing data from each accession were mapped to the corresponding *B. napus* ZS11 reference and further combined to build the A and C lineage SNP datasets based on a cross-ploidy pipeline (supplementary fig. S1, Supplementary Material online). For the resequenced accessions, the effective mapped read depth against the corresponding reference genome averaged 8×, with a range of 2.14× to 26.54× and the mapping rate averaged around 96.8% (supplementary table S2, Supplementary Material online). After variation discovery and filtering, variants were identified with relatively high quality, with 2,731,337 single nucleotide polymorphisms (SNPs) for the A lineage and 4,287,347 SNPs for the C lineage. Those variants were used for subsequent analyses.

### Origin and Phylogenetic History of *B. napus*

**Fig. 1.**
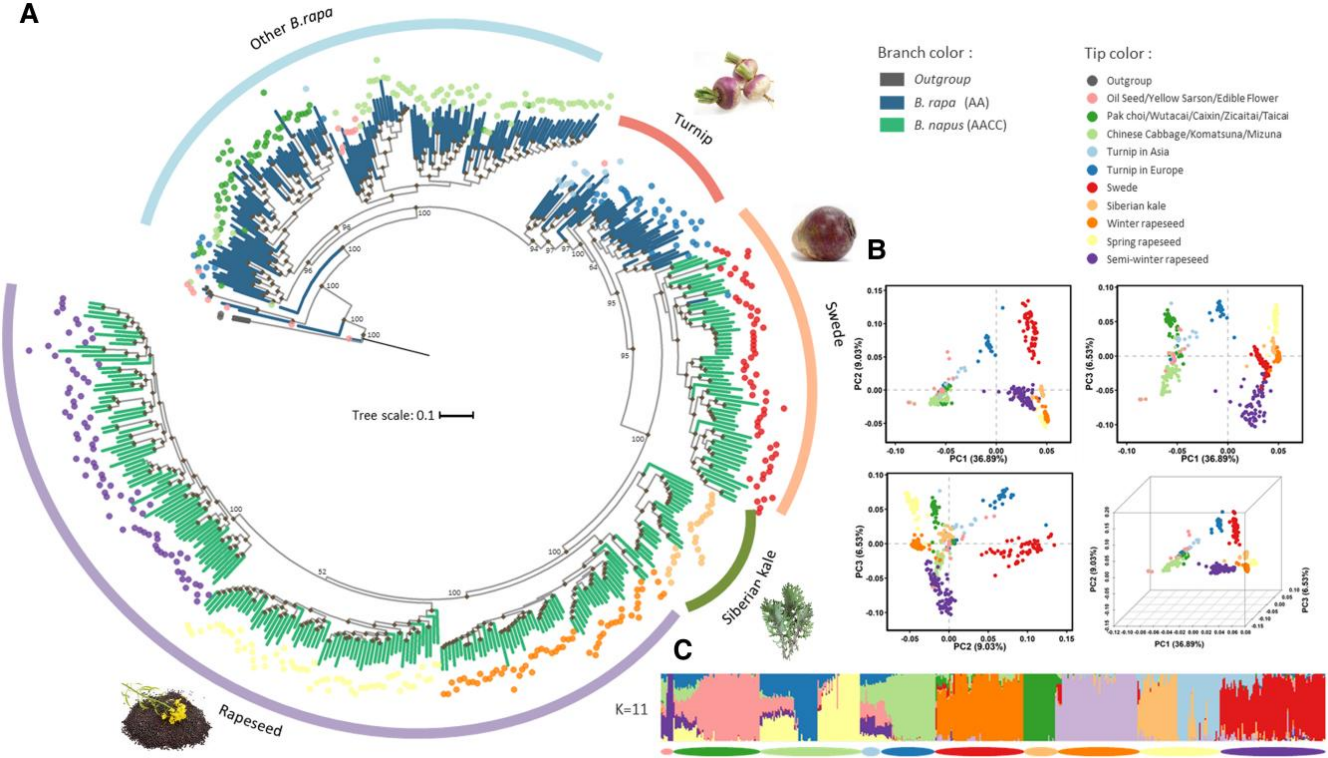
Phylogenetic relationship and population structure of the A lineage. (*A*) The phylogeny of the A lineage with *B. nigra* as the outgroup. The tip colors of the phylogeny indicate subspecies/morphotypes, whereas the branch colors denote the ploidy level. Branches with reliable bootstrap value (>70) are labeled with black point at the corresponding nodes. (*B*) PCA of the *B. rapa* and *B. napus* accessions. The proportions of variance explained by the top three principal components are presented in the axis labels. Colored points represent different morphotypes and are the same as the tip colors in the phylogeny. (*C*) Model-based Bayesian clustering performed with the number of ancestry kinship (*K*) set to 11. The different colors of vertical bar represent contributions to the K-groups. The colored segments of each horizontal bar indicate morphotypes.

**Fig. 2.**
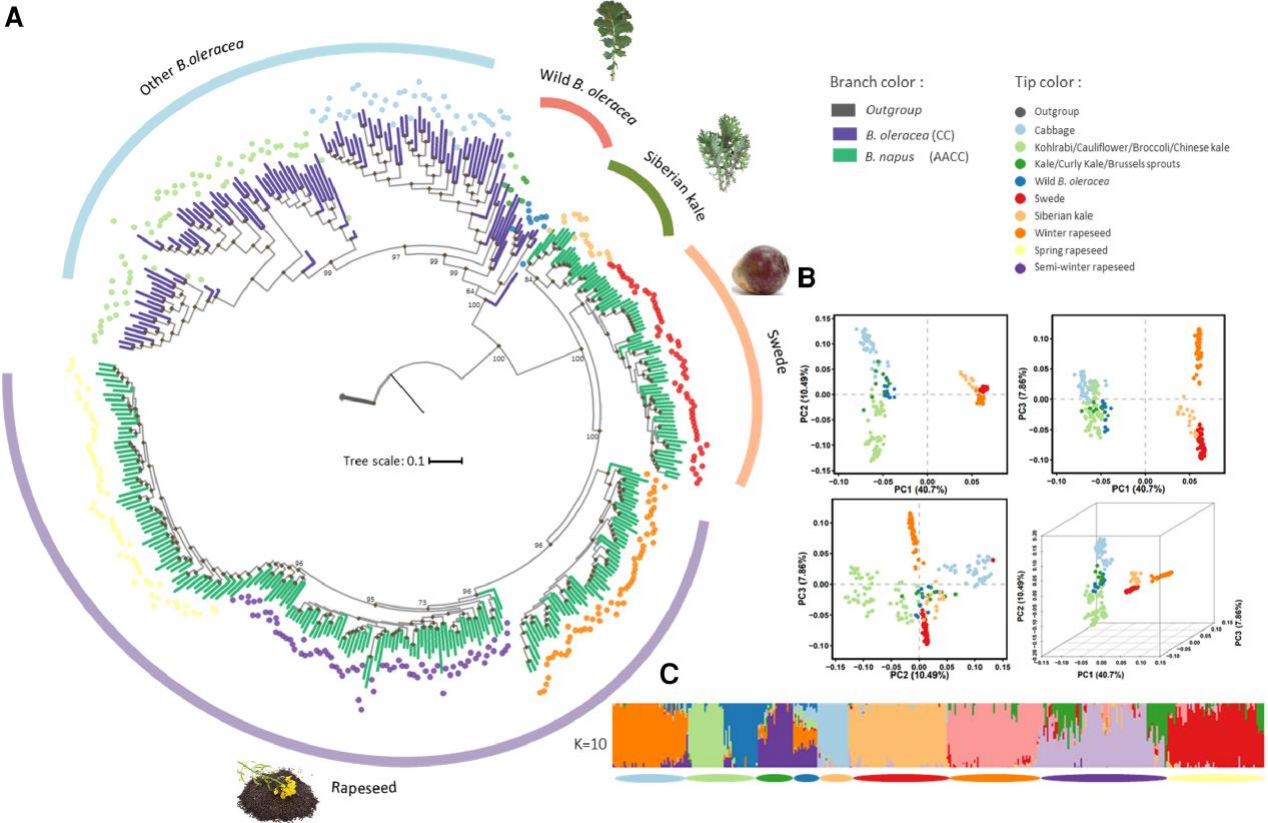
Phylogenetic relationship and population structure of the C lineage. (*A*) The phylogeny of the C lineage with *B. nigra* as the outgroup. The tip colors of the phylogeny indicate subspecies/morphotypes, whereas the branch colors denote the ploidy level. Branches with reliable bootstrap value (>70) are labeled with black point at the corresponding nodes. (*B*) PCA of the *B. oleracea* and *B. napus* accessions. The proportions of variance explained by the top three principal components are presented in the axis labels. Colored points represent different morphotypes and are the same as the tip colors in the phylogeny. (*C*) Model-based Bayesian clustering performed with the number of ancestry kinship (*K*) set to 10. The different colors of vertical bar represent contributions to the K-groups. The colored segments of each horizontal bar indicate morphotypes.

We first aim to understand the accurate origin, diploid progenitors, and phylogeny of *B. napus* as the fundamental first step in studying the adaptation of this polyploid species. SNPs were applied in multiple approaches to obtain robust phylogenetic histories for both the A and C lineages. With two accessions from *B. nigra* as the outgroup, maximum likelihood (ML) phylogenetic trees of the A and C lineages were constructed respectively, using 4-fold degenerate sites and best-fitting model selected by IQ-TREE (fig. 1*A* and 2*A*). To avoid potentially misleading interpretations of different topologies caused by conflicts from different datasets, we also randomly selected 200k SNPs from the A and C lineages for each phylogeny construction, and the results showed similar topology at most nodes (supplementary figs. S2 and S4, Supplementary Material online).

All of the *B. napus* accessions formed a single clade in both the A and C lineages from nuclear data, indicating *B. napus* was monophyletic and originated from a single hybridization event (figs. 1*A* and 2*A*). To further evaluate the hypothesis of the single origin of *B. napus*, we performed a comprehensive coalescent simulation analysis using fastsimcoal2, in which we compared multiple demographic models. We considered three major scenarios: 1) multiple origin from distinct diploid progenitors, 2) separate origin from the same diploid progenitors, and 3) single origin from the same diploid population. Our coalescent simulation results of both the A and C lineages provided consistent support for the scenario of a single tetraploid origin followed by admixture (supplementary fig. S6 and S7, Supplementary Material online). This result provides evidence in favor of the hypothesis that a single origin of *B. napus* was followed by extensive interspecies and intraspecies admixture.

The phylogeny of the A lineage revealed European turnip (*B. rapa* ssp. *rapa*) as the closest basal group to all *B. napus* (fig. 1*A*; supplementary fig. S2, Supplementary Material online). In line with this, principal component analysis (PCA) also located European turnips near the *B. napus* accessions (fig. 1*B*). These results are consistent with previous studies indicating European turnip as the direct progenitor of the *B. napus* A subgenome (Yang, et al. 2016). Among the *B. napus* clades, the phylogenetic clustering together with population structure resolved six genetic groups, which is highly congruent with different crop morphotypes and ecotypes (fig. 1*C*; supplementary fig. S3, Supplementary Material online). Interestingly, we found that the two subspecies, swede and Siberian kale, were the basal group to all the other oil-type rapeseeds, indicating these vegetable/feed morphotypes were the first crops to be domesticated. As for the common rapeseeds, the winter ecotype of rapeseeds was the original type, from which spring and semiwinter ecotypes evolved.

The phylogeny of the C lineage, with *B. nigra* as the outgroup, showed that wild *B. oleracea* accessions were closest to the *B. napus* clade (fig. 2*A*; supplementary figs. S4 and S5, Supplementary Material online). PCA of all accessions generally split the C lineage into two distant groups, illustrating divergence of C subgenomes between the two species *B. oleracea* and *B. napus*. Wild *B. oleracea* accessions were shown to have a closer position to Siberian kale and other *B. napus* groups (fig. 2*B*). This suggested that the direct donor of the C subgenome is most likely wild-type *B. oleracea*. Among the different *B. napus* morphotypes, Siberian kale and swede were also shown to be the first domesticated forms before oil-type rapeseeds, although the topology between the two subspecies is inconsistent with the A lineage. To mitigate the discordant position of swede and Siberian kale between the A and C subgenomes, we utilized the raw sequencing data from European turnip and wild *B. oleracea* accessions to represent the *in silico* tetraploid ancestry of *B. napus*. We randomly selected 150,000 SNPs from each genome to construct a phylogenetic history based on a total of 300,000 SNPs. Using the *in silico* ancestry accessions as the outgroup, the topology suggested tuber-type swede as the root clade in the phylogeny, which could support the hypothesis that the early domesticated forms of *B. napus* were used as root vegetables (supplementary fig. S8, Supplementary Material online).

**Fig. 3.**
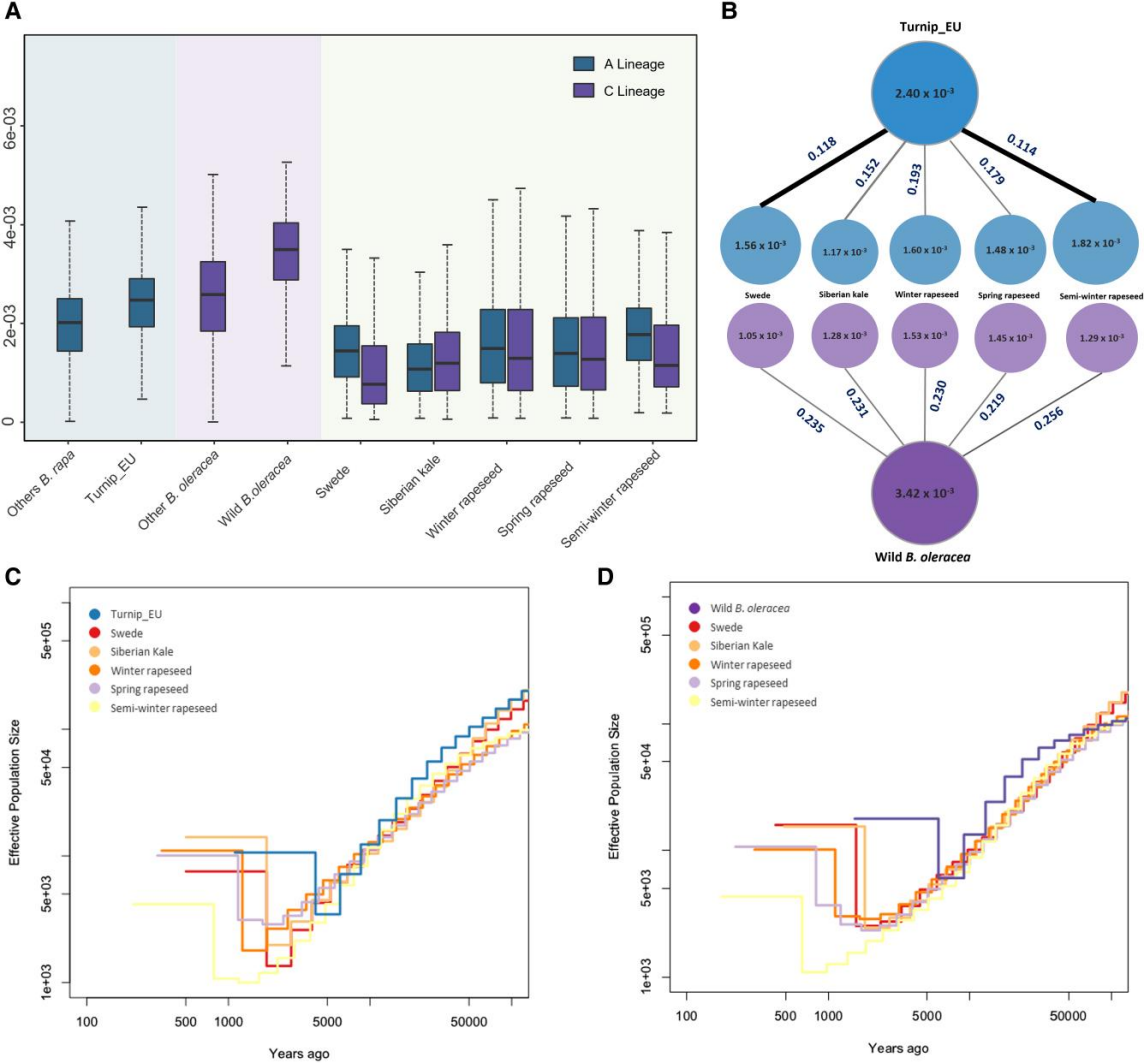
Genomic features of the A and C lineages. (*A*) The nucleotide diversity over 100-kb nonoverlapping windows in the A and C lineages. The middle line indicates the median value. The top and bottom of whisker denote the maximum and minimum value or the third quartile plus 1.5× the interquartile range (IQR). (*B*) Comparison of *F_st_* and nucleotide diversity between *B. napus* morphotypes and its direct progenitors. The value in each circle indicates the nucleotide diversity of the group, whereas the value on each line represents *F_st_* value between groups. (*C* and *D*) Demographic history of the A and C lineages inferred by MSMC model. Generation estimates were inferred by assuming that mutation rates were 1.5 × 10^−8^ per synonymous site per generation, respectively, and that the generation time was 1 year.

We next estimated historical changes in effective population size (*Ne*) using the multiple sequentially Markovian coalescent (MSMC). The overall *Ne* changes for different *B. napus* populations illustrated similar dynamics (fig. 3, *C* and *D*). All populations showed gradual decline in diversity since their divergence with progenitors around 5000–10,000 years ago. This period of declining *Ne* continued until the recent past, followed by rapid population expansion in recent 1,000 years.

### Genome-Wide Comparison of Genetic Diversity and Linkage Disequilibrium across A, C Lineages

To estimate and compare the genetic diversity across the A and C lineages, we conducted a comprehensive analysis of nucleotide diversity (*π*) and fixation statistics (*F_st_*) among different groups. The overall nucleotide diversity value for *B. napus* (*π_A_* = 1.55 × 10^−3^, *π_C_* = 1.37 × 10^−3^) was much lower compared with that of diploid *Brassica* vegetable crops *B. rapa* (2.17 × 10^−3^) and *B. oleracea* (2.98 × 10^−3^) (fig. 3*A*). Significant reduction of genetic diversity between *B. napus* and progenitors was observed, confirming the strong domestication and founder effect during recent polyploidization. Taking the two early diversified morphotypes swede and Siberian kale as example, the nucleotide diversity of their A subgenome captured only 50.8% from that of European turnip (one-tailed *t*-test, *P* < 2.2e−16) and for their C subgenome only 28.1% of that of wild *B. oleracea* (one-tailed *t*-test, *P* < 2.2e−16). Among *B. napus* populations, asymmetric distribution of nucleotide diversity between their A and C subgenomes with *π_A_* > *π_C_* was revealed in most groups, except for Siberian kale, which showed slightly higher *π_C_* than *π_A_*. Based on the A subgenome, semiwinter rapeseed and Siberian kale had the highest and lowest genetic diversity, respectively, whereas based on the C subgenome, winter rapeseed and swede showed the most and least genetic diversity. Interestingly, nucleotide diversity of the A subgenome in swede and semiwinter rapeseed groups was considerably higher than that of the C subgenome, being 1.88-fold and 1.33-fold higher, respectively.

The fixation index (*F_st_*) was further calculated on subgenome level to determine genetic differentiation between *B. napus* and progenitors. Generally, the *F_st_* value for *B. napus* with European turnip on the A subgenome was smaller than that with wild *B. oleracea* on the C subgenome (fig. 3*B*). Among *B. napus* populations, swede and semi-winter rapeseed showed the two lowest *F_st_* values with European turnip, compared with other groups. This corroborates with the finding of higher nucleotide diversity on A subgenome, indicating a closer genetic relationship of the A genomes of swede and semiwinter rapeseed with their A progenitors. The linkage disequilibrium (LD) decay (indicated by *r^2^*) of different groups across the A and C subgenomes generally showed that LD decay in progenitors was faster than in *B. napus* morphotypes, consistent with a bottleneck in both subgenomes (supplementary fig. S11, Supplementary Material online). The overall LD decay was stronger in the A subgenome than in the C subgenome. Among the *B. napus* populations, the level of LD varied, with Siberian kale showing the highest LD value and swede and spring rapeseed showing the lowest.

### Frequent Interploidy Introgression during *B. napus* Domestication

Since different populations of *B. napus* have largely been cultivated sympatrically with diploid progenitor populations during the species expansion and domestication, our prior hypothesis was that interploidy gene flow from diploids might play a significant role in the adaptation and worldwide expansion of different tetraploid *B. napus* populations. To test this hypothesis, we first systematically tested for the overall scale of introgression signatures in the A and C lineages by calculating ABBA-BABA statistics (Patterson's D) and *f_4_* admixture ratio (*f_4_*-ratio) statistics (Patterson, et al. 2012; Martin, et al. 2015). The D and *f_4_*-ratio statistics are commonly used to assess the evidence of gene flow and proportion of introgression between populations in genomic datasets. Both of them are based on examining excess patterns of shared alleles between potential P3 and either sister species P1 or P2 on a four-species tree model as ([P1, P2], P3, O), where O is the outgroup. The estimates of D and *f_4_*-ratio for the same P2–P3 species pairs displayed variation contingent on the distinct P1 populations, but overall trends were rather consistent. Hence, we used their maximal values to summarize the data and focus on the overall support for introgression between P2 and P3. We used *B. nigra* as the outgroup for all trios (combinations of three different populations) and estimated significance using a block jackknife approach.

**Fig. 4.**
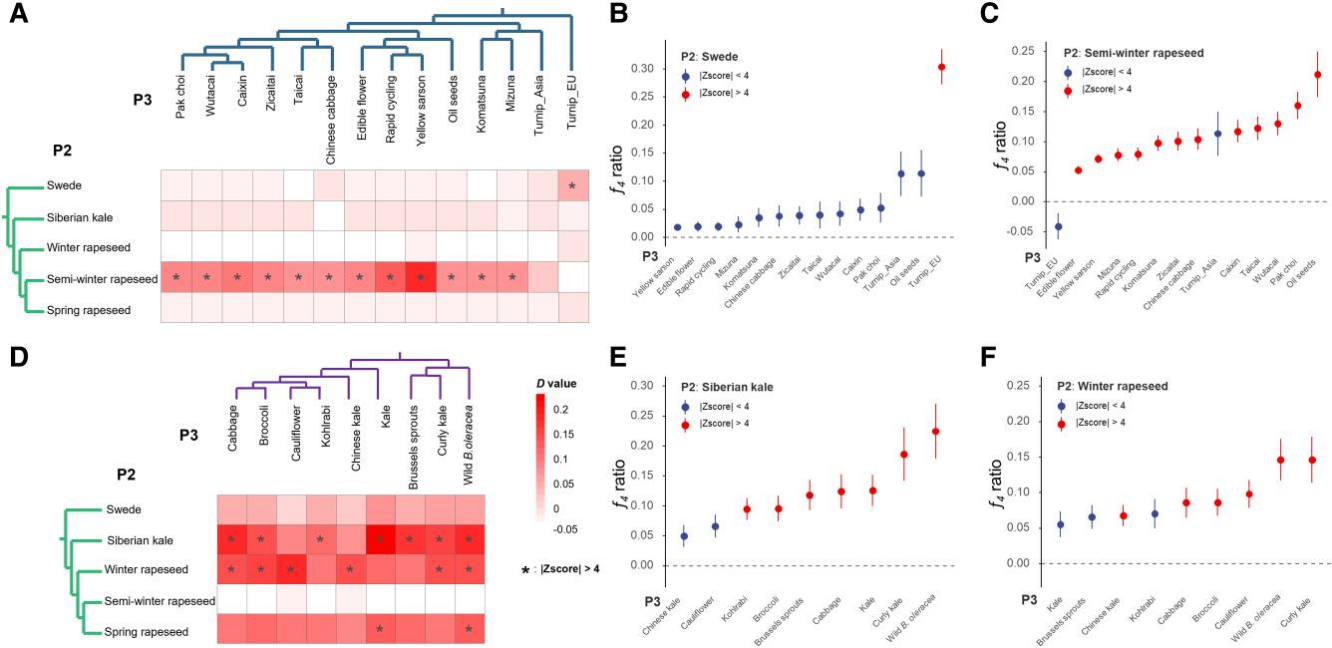
Frequent past interploidy introgression during *B. napus* domestication. (*A* and *D*) Heatmaps indicate maximum pairwise Patterson's *D* statistics measurements between pairs of morphotypes across all combinations in the A and C lineages. Asterisks indicate a significant value (|*Z*| > 4). (*B, C, E,* and *F*) *f_4_*-ratio statistics to test the proportion of interploidy introgression in specific groups. Colors of filled circles indicate a significant value threshold (|*Z*| > 4). The top and bottom whiskers correspond to 1 SE calculated across the A and C subgenomes using a weighted blocked jackknife. (Complete data sets are available in supplementary tables S3 and S4, Supplementary Material online.)

Overall, we tested a total of 140 trios in the A lineage, including 5 morphotypes of *B. napus* and 14 morphotypes of *B. rapa*. Of these trios, 48 had a significant *D* value at *Z*-score > 4, representing a total of 13 P2–P3 groups pairs. In the C lineage with 9 morphotypes of *B.* oleracea, 90 trios were tested, resulting in 15 P2–P3 groups pairs of D value to be significant. This provides strong evidence for pervasive historical introgression during *B. napus* expansion and domestication (fig. 4, *A* and *D*; supplementary figs. S12 and S13 and supplementary tables S3 and S4, Supplementary Material online). In the A lineage, two interploidy introgression events were found in *B. napus* populations (fig. 4*A*; supplementary table S3, Supplementary Material online**)**. The first one happened between tetraploid swede and diploid European turnip, which were largely cocultivated in European regions. The D statistics revealed a strong introgression signal in swede from European turnip compared with other *B. rapa* populations including Asian turnip. The *f_4_*-ratio further estimated around 30% proportion of introgressed genome between European turnip and swede, which is the highest value for introgression in *B. napus* (fig. 4*B*). Another large-scale introgression event happened between the semiwinter rapeseed and most Asian-cultivated *B. rapa* populations. Both D statistics and *f_4_*-ratio showed significant values for the introgression into semiwinter rapeseed from pak choi, caixin, taicai, komastsuna, and other Asian-derived *B. rapa* populations, with *f_4_*-ratio explaining 5–21% admixture proportion (fig. 4*C*). These introgression events reveal that when rapeseed was adopted from Europe into East Asia, intercrossing with cultivated diploid *B. rapa* accessions may have been applied to adapt rapeseed to the local climate with mild winters. In the C lineage, pervasive introgression events were also found from European-derived kale (*B. oleracea*) into Siberian kale and rapeseed ecotypes as they were largely distributed sympatrically (fig. 4*D*; supplementary table S4, Supplementary Material online). The proportion of introgression estimated by *f_4_*-ratio ranged from 6% to 22% (fig. 4, *E* and *F*).

We also conducted Treemix analysis to investigate potential gene flow events in both the A and C lineages (Pickrell and Pritchard 2012). The results indicate that intraspecies gene flow is widespread in both *B. rapa* and *B. oleracea*, consistent with previous studies (Mabry, et al. 2021; McAlvay, et al. 2021). Importantly, interploidy gene flow events were also detected in both lineages. In the A lineage, we observed gene flow from European turnip to swede and from different Asian cultivated morphotypes to semiwinter rapeseed (supplementary fig. S14, Supplementary Material online). Similarly, in the C lineage, we observed gene flow into different *B. napus* morphotypes from wild *B. oleracea*, kale, cabbage, and brussels sprouts, along with extensive intraspecies and interploidy introgressions (supplementary fig. S15, Supplementary Material online). Treemix analysis can differ compared with the D statistics (Patterson, et al. 2012), but these interploidy introgression signals were detected by both methods. In addition, given that homoeologous exchange (HE) has been demonstrated to be a widespread phenomenon in *B. napus*, we also examined the potential impact of HE on detecting introgression signals using the pipeline from a previous study (He, et al. 2017). Our findings show the high correlation coefficient between the D values obtained with and without excluding HE regions, suggesting that HE did not impact the identification of introgression events (supplementary fig. S16, Supplementary Material online). In summary, these results show that interploidy introgression was prevalent during *B. napus* domestication, with a likely role in adaptation to local environments and domestication for favorable traits.

### Identification of Interploidy Introgressions across the Genome and Their Characteristics

Given the prevalence of interploidy introgression from both progenitor populations into *B. napus*, we then investigated the potential adaptive importance of these interploidy introgression events during domestication. Putative introgression to chromosome regions for the trios with significant D values were localized through genome-wide calculation of both *f_d_* and *f_dM_* statistics (supplementary fig. S17, Supplementary Material online) (Patterson, et al. 2012; Martin, et al. 2015). Putative introgressed genomic regions were defined as the top *f_dM_* windows that summed to the genomic proportion estimated from the *f_4_-ratio* (supplementary figs. S18 and S19, Supplementary Material online).

**Fig. 5.**
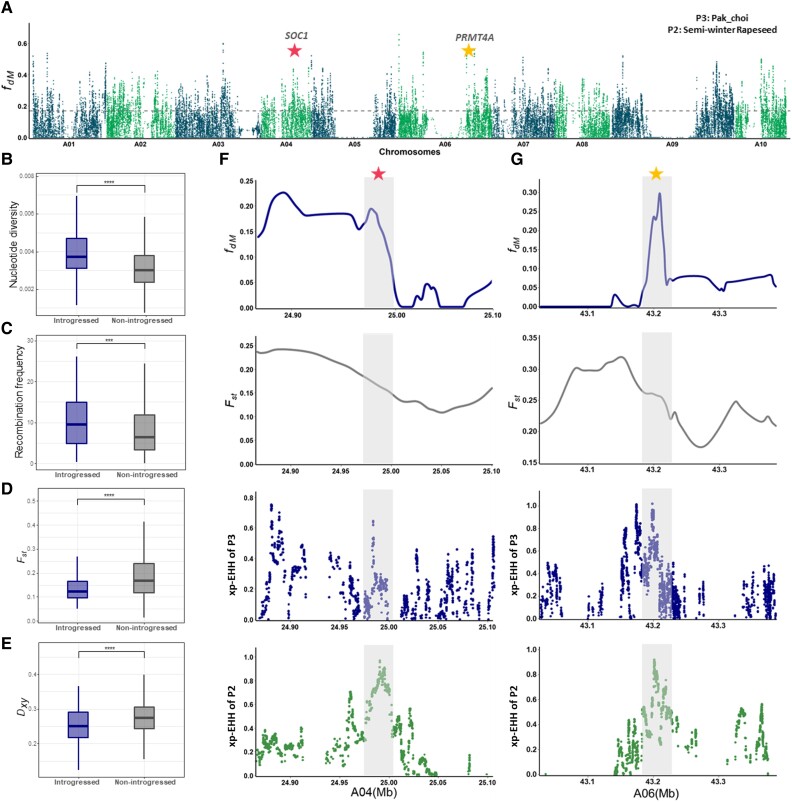
Genomic characteristics of the putative introgressed regions in semiwinter rapeseed. (*A*) Manhattan plot showing the *f_dM_* value across the A subgenome. The dashed line shows the cutoff value, calculated by highest x% of *f_dM_* values, where x was determined by the corresponding *f_4_*-ratio estimate. (*B, C, D,* and *E*) Diagrams indicate the comparison of nucleotide diversity, recombination rate, and genetic divergence (*F_st_* and *D_xy_*) between putative introgressed and nonintrogressed regions in semiwinter rapeseed. Mann–Whitney tests were used to assess significance between introgressed and nonintrogressed regions with asterisks indicating significance level. ***P < 0.001. (*F* and *G*) Magnification of the two representative adaptive introgressed regions showed by *f_dM_* and selective sweeps.

Various genomic characteristics were evaluated and compared between the putative introgressed and nonintrogressed chromosomal regions to assess the potential role of interploidy introgression. First, nucleotide diversity in the introgressed regions was significantly higher for most trios of *B. napus* populations in general, where proportion of increase ranged from 10.3% to 36.3%, with an average value of 23.2% (fig. 5*B*; supplementary fig. S20, Supplementary Material online), indicating that interploidy gene flow can increase genetic diversity, thereby alleviating the bottleneck effect resulting from allopolyploidy and speciation. Second, introgressed regions showed significantly lower genetic divergence, estimated by *F_st_* and *D_xy_*, between the donor and receptors, possibly explaining the lower genetic divergence of swede and semiwinter rapeseed with their A subgenome progenitors (fig. 5, *C* and *D*; supplementary figs. S21 and S22, Supplementary Material online). Third, introgressed regions were found to be confined to regions with high frequency of recombination, showing that high recombination regions tend to be more susceptible to introgression than regions with low recombination rates (fig. 5*E*; supplementary fig. S23, Supplementary Material online). In general, these genomic characteristics suggest some potential role of interploidy introgression for *B. napus*, possibly helping the various populations better adapt to local environment during domestication.

### Functional Significance of Interploidy Introgression during Domestication

We next selected representative interploidy introgression events and sought to determine their functional value during *B. napus* expansion and domestication. The first example is the introgression events from Asian-cultivated *B. rapa* morphotypes into semiwinter rapeseed (fig. 4, *A* and *C*). This ecotype has been recorded to be developed in East Asia from winter rapeseed over the last 200 years, where diploid *B. rapa* had already been domesticated into different vegetable morphotypes for thousands of years (Qi, et al. 2017). After localizing interploidy introgressions from *B. rapa* morphotypes into semiwinter rapeseed, 54–251 genomic regions were identified, according to different donor morphotypes, of which 2,201–6,950 genes were found to be potentially affected (supplementary fig. S18, Supplementary Material online). These genes were further examined using selective sweep analysis to determine their potential adaptive significance. We used both haplotype-based methods XP-EHH and XP-CLR to scan for candidate selective sweeps in semiwinter rapeseed with windows of the top 5% of maximum values considered as selection regions (supplementary fig. S24, Supplementary Material online). Two hundred fifty-three selective signals spanning 13.48 Mb were identified in both selective sweep methods, among which 67 signals overlapped with interploidy regions (significant according to Fisher's exact test, *P* < 0.001) (supplementary tables S5 and S6, Supplementary Material online). To gain further insight into the potential functions of overlapping selective introgression genes, we performed gene ontology (GO) enrichment analyses (supplementary fig. S25, Supplementary Material online). GO analyses for genes in those introgressed regions showed enriched terms involving regulation of developmental growth (GO: 0048638), regulation of flower development (GO: 0009909), seed growth (GO: 0080112), lipid storage (GO: 0019915), glucosinolate catabolic process (GO: 0019759), and response to gibberellin (GO: 0009739), revealing the potential adaptive significance for interploidy introgression.

We then conducted more detailed investigations of the potential connection between adaptive introgression and flowering time diversification, as it was an indispensable trait for semiwinter rapeseed to be successfully adapted from Europe into Asian regions. Compared with the rapeseed winter ecotype, semiwinter ecotypes have a weaker vernalization requirement as they are mainly grown in Asian regions with mild winters. We identified 631 orthologs of 306 *Arabidopsis thaliana* genes related to flowering time (FLOR-ID; Flowering Interactive Database) from the *B. napus* A subgenome (Bouché, et al. 2015). Pak choi morphotype was chosen as representative of the Asian *B. rapa* populations for localizing candidate regions because it showed one of the highest *f_4_-ratio* values in semiwinter rapeseed. We identified 105 flowering time-related genes that were among the 6,423 introgressed genes affected by pak choi (supplementary table S7, Supplementary Material online). Enrichment analysis further supported that introgressed regions were functionally enriched in flowering time genes (Fisher's exact test, *P* = 0.021, gene ratio: 105/6423, background ratio: 631/47,274), suggesting interploidy introgression played an important role for flowering time adaptation to the local environment. Those flowering genes in the introgressed regions included important vernalization-related genes (*SOC1*, *FRI*, *SVP*), circadian clock-related genes (*TOC4*, *PRR9*, *CKB4*), as well as photoperiod-related genes (*COL9, TSF*) and autonomous-related genes (*PRMT4A, FPF1*, *HUB1*). A specific example is given by *SOC1* (*SUPPRESSOR OF OVEREXPRESSION OF CO 1*, *BnaA04G0287900ZS*) on chromosome A04 and *PRMT4A* (*PROTEIN ARGININE METHYLTRANSFERASE 4A*, *BnaA06G0359200ZS*) on chromosome A06 (fig. 5*, F* and *G*). Both genes were found in introgressed regions, which showed lower genetic divergence between the donor pak choi and receptor semiwinter rapeseed. In addition, selection analysis on those shared regions revealed higher selective sweep signals in both receptor and donor populations, indicating a potentially important role for these two regions in domestication. Taken together, those results provide support for the role of interploidy introgression from locally adapted *B. rapa* morphotypes in flowering time adaptation to cultivation in Asian regions during the domestication of semiwinter rapeseed.

Similar interploidy selection significance was also found in European-cultivated *B. napus* morphotypes swede from diploid turnip. We identified 1,284 potential introgressed regions, spanning 32.4 Mb of the whole genome (fig. 4, *A* and *B*; supplementary fig. S18, Supplementary Material online). Selective sweep analysis further showed that 239 introgressed regions overlapped with selection regions, containing 1,204 genes (significant according to Fisher's exact test, *P* < 0.001) (supplementary fig. S26 and supplementary tables S8 and S9, Supplementary Material online). We next performed GO enrichment analyses of those potential adaptive introgressed genes, which identified significant overrepresentations in categories related to regulation of immune response (GO:0050776), photosynthesis (GO:0015979), sucrose metabolic process (GO:0005985), and other biological pathways (supplementary fig. S27, Supplementary Material online). Among the GO categories enriched, there were many relevant genes that may have functions during swede domestication. For example, sucrose transporter genes have been regarded as important meditators to transfer sugars from mature leaves to the swelling root. (Braun 2022). We found two representative genes *SWEET7* (*BnaA09T0254100ZS*) and *SUS3* (*BnaA09T0024200ZS*) on chromosome A09 that were affected by adaptive introgression in swede (supplementary fig. S28, Supplementary Material online). In regulation of immune response category, we identified the gene *WRKY33* (*BnaA05T0071200ZS*), whose orthologs are involved in multiple abiotic stresses (Wang, et al. 2018). Overall, our results suggest that introgression from diploid progenitors played an important role during domestication of the different *B. napus* morphotypes (supplementary fig. S29, Supplementary Material online).

## Discussion

### Origin and Evolutionary History of *B. napus* Populations

Species in the genus *Brassica* are known for their extreme diversity (Cheng, et al. 2014). Although several previous studies have reported the phylogeny and relationships of *B. napus*, its direct progenitors and intraspecies relationships have remained elusive (Li, et al. 2017; An, et al. 2019; Lu, et al. 2019; Wu, et al. 2019). In this study, to fully understand the phylogenetic relationship among the diverse morphotypes in *B. napus* (AACC) and its progenitors *B. rapa* (AA) and *B. oleracea* (CC), we generated whole-genome resequencing data together with public data, which represents the majority of morphotypes and ecotypes among these species. With *B. nigra* as the outgroup and multiple sources of variant datasets being used, our study has provided results about the phylogenetic relationship and origin of *B. napus,* as well as the adaptive importance of interploidy introgression from diploid species in the successful domestication of different *B. napus* morphotypes.

Understanding the origin and phylogeny of a species is always the fundamental first step in resolving other analyses and its better utilization in breeding. *Brassica napus* was thought to have originated from hybridization between *B. rapa* and *B. oleracea* less than 10,000 years ago (Chalhoub, et al. 2014; Lu, et al. 2019). Still, identification of specific A and C progenitor genotypes has remained elusive. In the present study, we used two different variant datasets (4-fold degenerate sites and randomly selected whole-genome variants) to build the ML phylogenetic tree for the A and C lineages, and both results showed similar topology (figs. 1*A*and 2*A*; supplementary figs. S2 and S4, Supplementary Material online). In combination with the Structure and PCA analysis, our phylogenetic results revealed that the A progenitor of *B. napus* was closest to the European turnip (*B. rapa*) and the C progenitor was closest to wild type *B. oleracea*. Our result of A progenitor is in line with several previous studies, which confirm that European turnip is the direct progenitor of the A subgenome of *B. napus* (Yang, et al. 2016; Lu, et al. 2019). As for the C progenitor, Lu et al. (2019) proposed that it evolved from the common ancestor of kohlrabi, cauliflower, broccoli, and Chinese kale, although they also discussed that broader sampling of *B. oleracea* accessions would be helpful to better understand the complex origin. In our study, owing to the extended sampling including the nonoil type subspecies of *B. napus* and wild *B. oleracea* accessions that were underrepresented in previous research, we identified wild *B. oleracea* as direct progenitor of the C subgenome. This result is in line with a prior finding based on AFLP data, which also put the wild-type *B. oleracea* at the closest position to all *B. napus* accessions (Allender and King 2010). Besides, given the domesticated status of the European turnip progenitor and the absence of wild populations of *B. napus*, it seems reasonable to assume that the original hybridization event probably took place in a cultivated rather than a natural environment, where wild *B. oleracea* plants were distributed sympatrically with European turnip populations. As European turnip was largely cultivated in European regions, and wild *B. oleracea* was found along the coastal regions in Europe (Mabry, et al. 2021; McAlvay, et al. 2021; Cai, et al. 2022), their vicinity enabled interspecific hybridization that finally led to the speciation of *B. napus*.

It is debated whether the allopolyploid species *B. napus* is the result of a single hybridization event or from multiple events. In our research, based on multiple datasets from nuclear genomes, we found that all *B. napus* morphotypes formed a single clade, supporting a monophyletic origin from a single hybridization event. In addition, coalescent simulations of population quartets from distinct diploid and tetraploid populations in both the A and C lineages consistently favor scenario of a single tetraploid origin, followed by interploidy and intraspecies admixture (supplementary figs. S6 and S7, Supplementary Material online**)**. Our result is congruent with several previous findings, which also support a monophyletic origin using nuclear genome data (An, et al. 2019; Lu, et al. 2019). On the other hand, several other studies based on chloroplast genome sequences concluded a potential multiorigin ancestry for the maternal *B. rapa* parent, with *B. napus* accessions grouping with different *B. rapa* morphotypes (Allender and King 2010; Li, et al. 2017). Given the prevalence of interploidy introgressions from different parental populations into *B. napus*, we think it is likely that those introgression events may have contributed to the diverse chloroplast genomes in the cases where *B. rapa* was the maternal parent.

The inconsistent topology of swede and Siberian kale we found in the A and C lineages leads to the question which morphotype evolved first during the intraspecific diversification in *B. napus*. To mitigate potential conflicts, we used datasets from combinations of European turnips and wild *B. oleracea* as the pseudo ancestry *B. napus*. We found that the root-vegetable swede was placed at the base position, supporting the hypothesis that swede was the first domesticated morphotype following *B. napus* speciation (supplementary fig. S8, Supplementary Material online). Furthermore, we mitigate the potential impact of introgression on topology inference by filtering SNPs from introgressed genomic regions. The discrepancy observed in the phylogenetic tree of the C lineage indicates that interploidy introgression from the C subgenome may have blurred inference on tree topology (supplementary figs. S9 and S10, Supplementary Material online). Intriguingly, we realized that the swollen hypocotyl-root vegetables in the *Brassica* genus including European turnip from *B. rapa* (AA), swede from *B. napus* (AACC), and root mustard from *B. juncea* (AABB) were all earliest morphotypes to emerge during their independent domestications (Yang, et al. 2018; McAlvay, et al. 2021). These morphotypes with enlarged root-hypocotyl tubers were cultivated before the oilseed morphotypes, possibly as the tubers provide starch and sugars as energy for human needs, whereas other crops may have been used as oil source. The swollen root-hypocotyl morphotypes of the two allopolyploid species *B. napus* and *B. juncea* represent interesting models for studying role of polyploidization and convergent evolution in the future.

### Contribution of Adaptive Introgressions from Diploids to the Success of Tetraploid *B. napus* Domestication

Hybridization across ploidy barriers can also break down species barriers, promote genetic variations in polyploids, and bring about adaptive traits (Marburger, et al. 2019; Novikova, et al. 2020; Edelman and Mallet 2021). Intriguingly, we found evidence for interploidy introgressions from various diploids into different *B. napus* morphotypes, which likely played an important role in adaptation to different environments and trait domestication by fueling adaptive genetic variations. In our study, both D statistics and *f_4_-ratio* statistics reveal that introgression is prevalent in *B. napus* and has shaped an appreciable proportion of extant genomes of different *B. napus* morphotypes (fig. 4). After comparing various genomic landscapes, introgressed regions were shown to have significantly higher genetic diversity and were associated with regions with higher recombination frequency (supplementary fig. S23, Supplementary Material online). This was in accordance with previous studies, which suggested that high recombinant regions tend to be more permissive to introgression because selection is more effective in separating neutral or beneficial alleles from deleterious alleles in areas with high recombination rates (Schumer, et al. 2018; Suarez-Gonzalez, et al. 2018). Although our dataset includes the majority of morphotypes and ecotypes in the three *Brassica* species, it is possible that some unsampled “ghost lineages,” including extinct ones, may have had direct interploidy introgression between the two diploid species and the allopolyploid species (Dagilis, et al. 2022; Tricou, et al. 2022).

We investigated whether those interploidy introgression events played a role in diversification and adaptation of semiwinter rapeseed and swede morphotypes to local environments. The semiwinter rapeseed was diversified from winter rapeseed based on our phylogeny and was recorded to be brought to Asian region to replace original oil-type *B. rapa* as new oilseed crop less than 200 years ago (Sun, et al. 2017). Swede represents a relatively old crop, which was largely cultivated in European regions (Gowers 2010). Both morphotypes have been cultivated sympatrically with various *B. rapa* and *B. oleracea* populations. Our introgression analysis reveals that there was indeed consistent interploidy gene flow from Asian cultivated *B. rapa* morphotypes (like pak choi, caixin, Chinese cabbage, etc.) into semiwinter rapeseed and from European turnips into swede. This could possibly explain the higher nucleotide diversity of their A subgenomes compared with the A genomes of the other morphotypes like spring and winter oilseed (fig. 3*A*). Such interploidy introgression can mitigate the severe bottleneck effect of allotetraploid *B. napus*. We identified several functional important genes, such as those related to flowering time diversification, in the introgressed regions, which colocated with strong selective sweep signals. Interestingly, in swede, the genes selected in introgressed regions related to sucrose transport, important for its fast-growing tubers, and immune response, likely related to their long growth and presence in soil. This study provides evidence that frequent interploidy introgressions from the congeneric diploids are important factors for successful domestication and adaptation of different *B. napus* morphotypes to a wide range of local environments.

## Materials and Methods

### Plant Materials and Sequencing

Seventeen accessions of swede/rutabaga (*B. napus* subsp. *rapifera*), 5 accessions of Siberian kale (*B. napus* subsp*. pabularia*), and 11 accessions of kale (*B. oleracea* subsp. *acephala*) and wild *B. oleracea* were collected for whole genome resequencing. Seeds were planted in the greenhouse during spring 2020 in order to confirm their morphotypes. Together with public data from previous studies (Cheng, et al. 2016; An, et al. 2019; Lu, et al. 2019; Wu, et al. 2019), the diversity panel used in this study contains 283 accessions of *B. napus*, 199 accessions of *B. rapa*, and 130 accessions of *B. oleracea*, representing most cultivar morphotypes and ecotypes of AA, CC, and AACC genomes. Besides, we also collected two accessions of *B. nigra* from public resources as the outgroup (Perumal, et al. 2020). Detailed information about morphotypes and ecotypes and geographic origins for each accession are shown in supplementary table S1, Supplementary Material online.

### DNA Extraction and Sequencing

Genomic DNA was extracted from leaf tissue for each accession using Qiagen DNeasy plant kit. Libraries with an insert size of 350 bp were constructed according to the standard manufacturer's protocol. Paired-end reads (2 × 150 bp) were generated using an Illumina NovaSeq 6,000 platform at Novogene-Tianjin. Samples were sequenced with an average depth of 15×. Reads with more than 5% of “N” bases or with more than half of bases having quality value less than Q20 were removed from the raw data.

### Variant Calling and Quality Control

The raw reads data from all accessions were filtered to remove adapters and low-quality bases using Trimmomatic (Bolger, et al. 2014) (version 0.38), with the parameters LEADING:3 TRAILING:3 SLIDINGWINDOW:5:15 MINLEN:50. The reference genomes *B. rapa* Chiifu (Zhang, Cai, et al. 2018) (AA, version 3), *B. oleracea* JZC (Cai, et al. 2020) (CC, version 2), and *B. napus* ZS11 (AACC) were selected (Song, et al. 2020). Filtered *B. napus* sequencing reads were aligned to AACC genome, and *B. rapa* and *B. oleracea* data were mapped to the corresponding ZS11 AA and CC subgenomes using BWA-MEM (Li and Durbin 2009) (version 0.7.12) with default parameters. The alignment results were sorted, and PCR duplicates were marked by Sambamba (Tarasov, et al. 2015) (version 0.7). Following Genome Analysis Toolkit (GATK) Best Practices (McKenna, et al. 2010), Variant calling for each accession was then carried out with the GATK HaplotypeCaller module and consolidated into a single GVCF file model, from which SNPs and InDels were finally identified using joint calling approach.

In order to remove false variants, only biallelic variants were retained, and the SNPs were further filtered with the following criteria: 1) quality filter: variants were filtered with parameters “QD < 2.0 || MQ < 40.0 || FS > 60.0 || SOR > 3.0 || MQRankSum < −12.5 || ReadPosRankSum < −8.0” using VariantFiltration module; 2) missing rate filter: variants with missing rate greater than 15% were removed; 3) depth filter: to avoid potential misalignment bias, variants showing ultrahigh or low mapping depth were filtered out using VCFtools (Danecek, et al. 2011) (version 0.1.16) with parameters “–min-meanDP 3, –max-meanDP 40, –minDP 3, –maxDP 40”; and 4) minor allele count filter: to keep the statistical power for population statistics, rare SNPs were filtered out using “–mac 3” in VCFtools.

### Detection of Syntenic Sites for A and C Lineages

Given the different ploidy level across species, we used a cross-ploidy pipeline, and only syntenic or conserved SNPs from the A and C subgenomes were retained to build A and C variants lineages (supplementary fig. S1, Supplementary Material online). NUCmer module from MUMmer4 (Marçais, et al. 2018) (version 4.0.2) was used to align the two subgenomes in the A and C lineages, respectively, with default parameters, following which the delta-filter module was used to obtain one-to-one syntenic blocks in the alignment results with parameters “-r –q.” Finally, SNPs retrieved from the A subgenome of *B. rapa* and *B. napus* were combined and filtered by syntenic blocks. These SNPs were defined as the A lineage variants. SNPs retrieved from the *B. oleracea* and *B. napus* C subgenome were similarly combined and filtered to represent the C lineage variants.

### Phylogenetic Inference and Population Structure

To construct a ML phylogenetic tree, we used 4-fold degenerate sites SNPs to reduce the potential influence of natural or artificial selection. Besides, we also randomly select 200k SNPs from corresponding subgenomes for each phylogeny construction. Phylogenetic tree for each lineage was reconstructed using IQ-TREE (Minh, et al. 2020) (version 2.0.3), based on the best fitting model (TVM + R10), determined by the Bayesian information criterion. Bootstrap values were calculated using the ultrafast bootstrap method (UFboot) with 1,000 replicates. The output tree was then plotted and visualized by R package ggtree (Yu 2020) (version 3.0.2) with two accessions from *B. nigra* as the outgroup.

PCA was performed by PLINK (Purcell, et al. 2007) (version 1.90b4). The top three principal components were used and plotted in R. We then used fastSTRUCTURE (Raj, et al. 2014) to perform population structure analysis for each lineage, with the number of clusters (K) been set from 2 to 20. The optimal *K*, which maximizes the marginal likelihood, was evaluated by the script chooseK.py. The output structure results were further visualized using pophelper (Francis 2017) package in R.

### Estimation of Demographic History

We used MSMC2 software (Malaspinas, et al. 2016) based on MSMC approach to estimate historical patterns of effective population sizes for each lineage. We selected 10 accessions from each population with high mapping depth. The mask files for the genome were calculated using the bamCaller.py script of msmc-tools, where genomic sites having less than average coverage were filtered out. In addition, the software SNPable (http://lh3lh3.users.sourceforge.net/snpable.shtml) was used to create a mappability mask for the reference genome. Sites with the majority of overlapping 100-mers without mismatch were defined as the SNPable sites and used for the following MSMC analysis. We then estimated effective population size using MSMC2 with the pattern parameters “1 × 2 + 25 × 1 + 1 × 2 + 1 × 3.” To convert the coalescent scaled time to absolute time in years, we used a mutation rate of upper 1.5e−8 and lower 9e−9 from previous research and a generation time of 1 year. The output files were then plotted and visualized in R software.

We also performed a comparative analysis of demographic models and estimated parameters utilizing the coalescent simulation software, fastsimcoal2, to determine whether *B. napus* had a single or multiple origins, as established in the previous pipeline (Monnahan, et al. 2019). Population quartets consisting of representatives from both parental diploid populations and tetraploids were utilized for this analysis. The models were constructed with varying topologies and accounted for the presence or absence of migration (admixture) events. Each model was then fitted to a multidimensional site frequency spectrum calculated from the 4-fold degenerate SNP data. By using these methods, we aimed to discern the most plausible demographic scenario for the origin of *B. napus*. For each demographic scenario and population quartet, we conducted 30 independent fastsimcoal2 runs. We then extracted the best likelihood partition for each fastsimcoal2 run, calculated the Akaike Information Criterion (AIC), and aggregated the AIC values across the 30 independent fastsimcoal2 runs over the scenarios tested within each population quartet. The scenario exhibiting a consistently lower AIC value (ΔAIC > 2) within a particular population quartet was considered to be the preferred model.

### Genetic Diversity and Divergence Analysis

We calculated the nucleotide diversity for each subgenome in a window of nonoverlapping 10 kb using VCFtools. Weir and Cockerham's *F_ST_* value and *D_xy_* value between two populations were calculated using VCFtools with a window size 10 kb. Average *F_ST_* values were then calculated to represent the mean *F_ST_* value between two populations. Linkage disequilibrium (*r^2^*) was calculated by PopLDdecay (Zhang, Dong, et al. 2018) (version 3.41) with parameters “-MaxDist 1000 -Het 0.1 -Miss 0.1.”

### Historical Recombination Rate Estimation

SNPs for each lineage were firstly phased using Beagle (Browning, et al. 2018) (version 4.1) with default parameter settings and 30 iterations in a 50 kb sliding window. Then, FastEPRR (Gao, et al. 2016) (version 2.0) was employed for estimating population recombination rate, which is denoted as $\rho =4N_{e}r$ (*N* is the effective population size and *r* is the recombination rate of the window), with a 50 kb nonoverlapping window size.

### Detection of Past Introgression

To estimate the signals of past introgression among different morphotypes in the A and C lineages, the overall Patterson's *D* statistics (ABBA/BABA) (Patterson, et al. 2012) was used to examine introgression sites with a defined tree topology for the four groups as ([{P1, P2}, P3], O). For each lineage, two accessions from *B. nigra* were used as the outgroup (O) to evaluate whether P1 or P2 shared more alleles with a potential introgression P3 than with outgroup. *D* statistics for all trios of population from the A and C lineages were calculated using Dtrios module in Dsuite (Malinsky, et al. 2021) (version 0.4) with default parameters. *D* statistics significantly differing from 0 indicates introgression between P1 and P3 (*D* < 0) or between P2 and P3 (*D* > 0). The overall *f_4_* admixture ratio for all trios, which estimates the proportion of introgressed genome from donor, was calculated using admixr (Petr, et al. 2019) (version 0.9.1). The significance of the *D* statistics and standard error were calculated using a block jackknifing approach. To avoid repeating data and focus on the main evidence of gene flow between P2 and P3, we used the highest estimates of D and f4-ratio for each P2–P3 pair for the following analysis.

To further locate the introgressed regions across the genome for each trio, we used the *f_d_* and its modified statistics *f_dM_* using a sliding window of 10 kb with steps of 1 kb throughout the genome (Martin, et al. 2015). We defined the putative introgressed regions as windows with highest x% of *f_dM_* values, where x was determined for each trio by the proportion of introgression estimated by the *f_4_* ratio statistics, following the approach used by (Morales-Cruz, et al. 2021). Filtered windows were further merged to represent the final introgressed regions.

We also applied Treemix (version 1.3) to infer migration events and population relatedness in both the A and C lineages (Pickrell and Pritchard 2012). To reduce the impact of highly linked genomic regions, we employed a 50 kb sliding window (with steps of 10 SNPs) to scan the entire genome and then eliminated any SNPs that had a strong association (*r*^2^ > 0.2). We used the LD-pruned sites to construct a tree without any migration events and then used this tree as the basis for migration models. We built admixture trees with 1–15 migration events and evaluated the model fit for each migration event by estimating the proportion of variance explained by each migration model among all the subgroups. The numbers of migration edges were estimated aided by the optM (v0.1.5) package (Fitak 2021). The resulting gene flow and migration events were further visualized in R.

### Relationship between Introgression and Genetic Characteristics

To characterize the relationship between genomic features and introgression, we compared the nucleotide diversity, *F_st_*, *D_xy_*, and recombination frequency between introgressed regions and other genomic regions. We estimated those genomic features in the same window of *f_dM_* statistics using the software described before. The boxplot of the comparison for each genomic pattern was plotted using ggplot2 in R.

### Detection of Selective Sweeps

A cross-population composite likelihood ratio test (XP-CLR) was used to identify selective sweeps regions in different morphotypes based on the SNPs with less than 10% missing data (Chen, Patterson, et al. 2010). The XP-CLR score between two populations was calculated using parameters of -w1 0.005 500 10,000 -p1 0.95 for each chromosome. Genetic distances were estimated according to physical distances in a previous high-density genetic map (Yang, et al. 2017). The mean XP-CLR score was calculated using 100 kb sliding windows with a 10 kb step size. The R package GenWin was used for the normalization and detecting the boundary of genomic regions with smoothness = 1,000 and method = 4. Moreover, we also estimated the cross-population extended haplotype homozygosity (XP-EHH) for each population using Selscan, after filtering all missing data, with 50 kb sliding windows and 25 kb step size (Szpiech and Hernandez 2014). Sliding windows with average XP-CLR scores and XP-EHH scores higher than 95th percentile were selected as significant windows.

### GO Enrichment Analysis

GO terms for the *B. napus* genes were assigned based on syntenic relationship with Arabidopsis genes detected by Synorths (Cheng, et al. 2012). To identify the biological processes of genes in introgressed regions and selective sweeps regions, GO analysis was performed with ClusterProfiler (Wu, et al. 2021) (version 4.0). Enrichment significance was analyzed with Fisher's exact test. *P* values were further corrected for multiple comparisons using the method of Bonferroni.

## Supplementary Material

msad199_Supplementary_Data

## Data Availability

All data needed to evaluate the conclusions in the paper are present in the Supplementary material. The newly generated genome sequencing data of the samples produced in this study have been deposited in the Sequence Read Archive (SRA) under the BioProject accession number PRJNA888419. The variation datasets for SNPs in this work are available through BRAD website (http://brassicadb.cn). The custom code associated with this project is available at https://github.com/wang-tianpeng/Bnapus_interploidy_domestication.

## References

[msad199-B1] Allender CJ , King GJ. 2010. Origins of the amphiploid species *Brassica napus* L. investigated by chloroplast and nuclear molecular markers. BMC Plant Biol. 10(1):54. 20350303 10.1186/1471-2229-10-54 PMC2923528

[msad199-B2] An H , Qi X, Gaynor ML, Hao Y, Gebken SC, Mabry ME, McAlvay AC, Teakle GR, Conant GC, Barker MS, et al 2019. Transcriptome and organellar sequencing highlights the complex origin and diversification of allotetraploid *Brassica napus*. Nat Commun. 10(1):2878. 31253789 10.1038/s41467-019-10757-1 PMC6599199

[msad199-B3] Arrigo N , Barker MS. 2012. Rarely successful polyploids and their legacy in plant genomes. Curr Opin Plant Biol. 15(2):140–146. 22480430 10.1016/j.pbi.2012.03.010

[msad199-B4] Baack EJ , Rieseberg LH. 2007. A genomic view of introgression and hybrid speciation. Curr Opin Genet Dev. 17(6):513–518. 17933508 10.1016/j.gde.2007.09.001 PMC2173880

[msad199-B5] Bolger AM , Lohse M, Usadel B. 2014. Trimmomatic: a flexible trimmer for illumina sequence data. Bioinformatics. 30(15):2114–2120. 24695404 10.1093/bioinformatics/btu170 PMC4103590

[msad199-B6] Bouché F , Lobet G, Tocquin P, Périlleux C. 2015. FLOR-ID: an interactive database of flowering-time gene networks in *Arabidopsis thaliana*. Nucleic Acids Res. 44(D1):D1167–D1171. 26476447 10.1093/nar/gkv1054 PMC4702789

[msad199-B7] Braun DM . 2022. Phloem loading and unloading of sucrose: what a long, strange trip from source to sink. Annu Rev Plant Biol. 73:553–584. 35171647 10.1146/annurev-arplant-070721-083240

[msad199-B8] Browning BL , Zhou Y, Browning SR. 2018. A one-penny imputed genome from next-generation reference panels. Am J Hum Genet. 103(3):338–348. 30100085 10.1016/j.ajhg.2018.07.015 PMC6128308

[msad199-B9] Cai C , Bucher J, Bakker FT, Bonnema G. 2022. Evidence for two domestication lineages supporting a middle-eastern origin for *Brassica oleracea* crops from diversified kale populations. Hortic Res. 9:uhac033. 35184188 10.1093/hr/uhac033 PMC8976692

[msad199-B10] Cai X , Wu J, Liang J, Lin R, Zhang K, Cheng F, Wang X. 2020. Improved *Brassica oleracea* JZS assembly reveals significant changing of LTR-RT dynamics in different morphotypes. Theor Appl Genet. 133(11):3187–3199. 32772134 10.1007/s00122-020-03664-3

[msad199-B11] Cenci A , Sardos J, Hueber Y, Martin G, Breton C, Roux N, Swennen R, Carpentier SC, Rouard M. 2020. Unravelling the complex story of intergenomic recombination in ABB allotriploid bananas. Ann Bot. 127(1):7–20. 10.1093/aob/mcaa032 PMC7750727 32104882

[msad199-B12] Chalhoub B , Denoeud F, Liu S, Parkin IA, Tang H, Wang X, Chiquet J, Belcram H, Tong C, Samans B, et al 2014. Early allopolyploid evolution in the post-Neolithic *Brassica napus* oilseed genome. Science. 345(6199):950–953. 25146293 10.1126/science.1253435

[msad199-B13] Chapman MA , Abbott RJ. 2010. Introgression of fitness genes across a ploidy barrier. New Phytol. 186(1):63–71. 19912548 10.1111/j.1469-8137.2009.03091.x

[msad199-B14] Chatterjee D , Banga S, Gupta M, Bharti S, Salisbury PA, Banga SS. 2016. Resynthesis of *Brassica napus* through hybridization between B. juncea and B. carinata. Theo Appl Genet. 129(5):977–990. 10.1007/s00122-016-2677-3 26849238

[msad199-B15] Chen H , Patterson N, Reich D. 2010. Population differentiation as a test for selective sweeps. Genome Res. 20(3):393–402. 20086244 10.1101/gr.100545.109 PMC2840981

[msad199-B16] Chen S , Zou J, Cowling WA, Meng J. 2010. Allelic diversity in a novel gene pool of canola-quality *Brassica napus* enriched with alleles from *B. rapa* and *B. carinata*. Crop Pasture Sci. 61(6):483–492.

[msad199-B17] Cheng F , Liang J, Cai C, Cai X, Wu J, Wang X. 2017. Genome sequencing supports a multi-vertex model for Brassiceae species. Curr Opin Plant Biol. 36:79–87. 28242534 10.1016/j.pbi.2017.01.006

[msad199-B18] Cheng F , Sun R, Hou X, Zheng H, Zhang F, Zhang Y, Liu B, Liang J, Zhuang M, Liu Y, et al 2016. Subgenome parallel selection is associated with morphotype diversification and convergent crop domestication in *Brassica rapa* and *Brassica oleracea*. Nat Genet. 48(10):1218–1224. 27526322 10.1038/ng.3634

[msad199-B19] Cheng F , Wu J, Fang L, Wang X. 2012. Syntenic gene analysis between *Brassica rapa* and other Brassicaceae species. Front Plant Sci. 3:198. 22969786 10.3389/fpls.2012.00198 PMC3430884

[msad199-B20] Cheng F , Wu J, Wang X. 2014. Genome triplication drove the diversification of *Brassica* plants. Hortic Res. 1(1):14024. 26504539 10.1038/hortres.2014.24 PMC4596316

[msad199-B21] Dagilis AJ , Peede D, Coughlan JM, Jofre GI, D'Agostino ERR, Mavengere H, Tate AD, Matute DR. 2022. A need for standardized reporting of introgression: insights from studies across eukaryotes. Evol Lett. 6(5):344–357. 36254258 10.1002/evl3.294 PMC9554761

[msad199-B22] Danecek P , Auton A, Abecasis G, Albers CA, Banks E, DePristo MA, Handsaker RE, Lunter G, Marth GT, Sherry ST. 2011. The variant call format and VCFtools. Bioinformatics. 27(15):2156–2158. 21653522 10.1093/bioinformatics/btr330 PMC3137218

[msad199-B23] Dempewolf H , Baute G, Anderson J, Kilian B, Smith C, Guarino L. 2017. Past and future use of wild relatives in crop breeding. Crop Sci. 57(3):1070–1082.

[msad199-B24] De Queiroz K . 2007. Species concepts and species delimitation. Syst Biol. 56(6):879–886. 18027281 10.1080/10635150701701083

[msad199-B25] Edelman NB , Mallet J. 2021. Prevalence and adaptive impact of introgression. Annu Rev Genet. 55:265–283. 34579539 10.1146/annurev-genet-021821-020805

[msad199-B26] Fitak RR . 2021. Optm: estimating the optimal number of migration edges on population trees using Treemix. Biol Methods Protocols. 6(1):bpab017. 10.1093/biomethods/bpab017 PMC8476930 34595352

[msad199-B27] Francis RM . 2017. Pophelper: an R package and web app to analyse and visualize population structure. Mol Ecol Resour. 17(1):27–32. 26850166 10.1111/1755-0998.12509

[msad199-B28] Gao F , Ming C, Hu W, Li H. 2016. New software for the fast estimation of population recombination rates (FastEPRR) in the genomic era. G3 (Bethesda). 6(6):1563–1571. 27172192 10.1534/g3.116.028233 PMC4889653

[msad199-B29] Gowers S . 2010. Swedes and turnips. In: Bradshaw JE, editor. Root and tuber crops. New York, NY: Springer New York. p. 245–289.

[msad199-B30] Han TS , Wu Q, Hou XH, Li ZW, Zou YP, Ge S, Guo YL. 2015. Frequent introgressions from diploid species contribute to the adaptation of the tetraploid Shepherd's Purse (*Capsella Bursa-pastoris*). Mol Plant. 8(3):427–438. 25661060 10.1016/j.molp.2014.11.016

[msad199-B31] Havlickova L , He Z, Wang L, Langer S, Harper AL, Kaur H, Broadley MR, Gegas V, Bancroft I. 2018. Validation of an updated Associative Transcriptomics platform for the polyploid crop species *Brassica napus* by dissection of the genetic architecture of erucic acid and tocopherol isoform variation in seeds. Plant J. 93(1):181–192. 29124814 10.1111/tpj.13767 PMC5767744

[msad199-B32] He F , Pasam R, Shi F, Kant S, Keeble-Gagnere G, Kay P, Forrest K, Fritz A, Hucl P, Wiebe K, et al 2019. Exome sequencing highlights the role of wild-relative introgression in shaping the adaptive landscape of the wheat genome. Nat Genet. 51(5):896–904. 31043759 10.1038/s41588-019-0382-2

[msad199-B33] He Z , Wang L, Harper AL, Havlickova L, Pradhan AK, Parkin IAP, Bancroft I. 2017. Extensive homoeologous genome exchanges in allopolyploid crops revealed by mRNAseq-based visualization. Plant Biotechnol J. 15(5):594–604. 27808473 10.1111/pbi.12657 PMC5399007

[msad199-B34] Heslop-Harrison P . 2013. Genetics, genomics and breeding of oilseed Brassicas. Ann Bot. 112(3):vi–vi.

[msad199-B35] Hilu KW . 1993. Polyploidy and the evolution of domesticated plants. Am J Bot. 80(12):1494–1499.

[msad199-B36] Janzen GM , Wang L, Hufford MB. 2019. The extent of adaptive wild introgression in crops. New Phytol. 221(3):1279–1288. 30368812 10.1111/nph.15457

[msad199-B37] Kim M , Cui M-L, Cubas P, Gillies A, Lee K, Chapman MA, Abbott RJ, Coen E. 2008. Regulatory genes control a key morphological and ecological trait transferred between species. Science. 322(5904):1116–1119. 19008450 10.1126/science.1164371

[msad199-B38] Landis JB , Soltis DE, Li Z, Marx HE, Barker MS, Tank DC, Soltis PS. 2018. Impact of whole-genome duplication events on diversification rates in angiosperms. Am J Bot. 105(3):348–363. 29719043 10.1002/ajb2.1060

[msad199-B39] Leebens-Mack JH , Barker MS, Carpenter EJ, Deyholos MK, Gitzendanner MA, Graham SW, Grosse I, Li Z, Melkonian M, Mirarab S, et al 2019. One thousand plant transcriptomes and the phylogenomics of green plants. Nature. 574(7780):679–685. 31645766 10.1038/s41586-019-1693-2 PMC6872490

[msad199-B40] Leijten W , Koes R, Roobeek I, Frugis G. 2018. Translating flowering time from *Arabidopsis thaliana* to Brassicaceae and Asteraceae crop species. Plants. 7(4):111. 30558374 10.3390/plants7040111 PMC6313873

[msad199-B41] Li H , Durbin R. 2009. Fast and accurate short read alignment with burrows–wheeler transform. Bioinformatics. 25(14):1754–1760. 19451168 10.1093/bioinformatics/btp324 PMC2705234

[msad199-B42] Li P , Zhang S, Li F, Zhang S, Zhang H, Wang X, Sun R, Bonnema G, Borm TJA. 2017. A phylogenetic analysis of chloroplast genomes elucidates the relationships of the six economically important *Brassica* species comprising the triangle of U. Front Plant Sci. 8:111. 28210266 10.3389/fpls.2017.00111 PMC5288352

[msad199-B43] Liu S , Zhang L, Sang Y, Lai Q, Zhang X, Jia C, Long Z, Wu J, Ma T, Mao K, et al 2022. Demographic history and natural selection shape patterns of deleterious mutation load and barriers to introgression across *Populus* genome. Mol Biol Evol. 39(2):msac008. 35022759 10.1093/molbev/msac008 PMC8826634

[msad199-B44] Lu K , Wei L, Li X, Wang Y, Wu J, Liu M, Zhang C, Chen Z, Xiao Z, Jian H, et al 2019. Whole-genome resequencing reveals *Brassica napus* origin and genetic loci involved in its improvement. Nat Commun. 10(1):1154. 30858362 10.1038/s41467-019-09134-9 PMC6411957

[msad199-B45] Mabry ME , Turner-Hissong SD, Gallagher EY, McAlvay AC, An H, Edger PP, Moore JD, Pink DAC, Teakle GR, Stevens CJ, et al 2021. The evolutionary history of wild, domesticated, and feral *Brassica oleracea* (Brassicaceae). Mol Biol Evol. 38(10):4419–4434. 34157722 10.1093/molbev/msab183 PMC8476135

[msad199-B46] Malaspinas A-S , Westaway MC, Muller C, Sousa VC, Lao O, Alves I, Bergström A, Athanasiadis G, Cheng JY, Crawford JE, et al 2016. A genomic history of aboriginal Australia. Nature. 538(7624):207–214. 27654914 10.1038/nature18299 PMC7617037

[msad199-B47] Malinsky M , Matschiner M, Svardal H. 2021. Dsuite—fast D-statistics and related admixture evidence from VCF files. Mol Ecol Resour. 21(2):584–595. 33012121 10.1111/1755-0998.13265 PMC7116594

[msad199-B48] Marburger S , Monnahan P, Seear PJ, Martin SH, Koch J, Paajanen P, Bohutínská M, Higgins JD, Schmickl R, Yant L. 2019. Interspecific introgression mediates adaptation to whole genome duplication. Nat Commun. 10(1):5218. 31740675 10.1038/s41467-019-13159-5 PMC6861236

[msad199-B49] Marçais G , Delcher AL, Phillippy AM, Coston R, Salzberg SL, Zimin A. 2018. MUMmer4: a fast and versatile genome alignment system. PLoS Comput Biol. 14(1):e1005944. 29373581 10.1371/journal.pcbi.1005944 PMC5802927

[msad199-B50] Martin SH , Davey JW, Jiggins CD. 2015. Evaluating the use of ABBA-BABA statistics to locate introgressed loci. Mol Biol Evol. 32(1):244–257. 25246699 10.1093/molbev/msu269 PMC4271521

[msad199-B51] Mason AS , Batley J. 2015. Creating new interspecific hybrid and polyploid crops. Trends Biotechnol. 33(8):436–441. 26164645 10.1016/j.tibtech.2015.06.004

[msad199-B52] McAlvay AC , Ragsdale AP, Mabry ME, Qi X, Bird KA, Velasco P, An H, Pires JC, Emshwiller E. 2021. *Brassica rapa* domestication: untangling wild and feral forms and convergence of crop morphotypes. Mol Biol Evol. 38(8):3358–3372. 33930151 10.1093/molbev/msab108 PMC8321528

[msad199-B53] McKenna A , Hanna M, Banks E, Sivachenko A, Cibulskis K, Kernytsky A, Garimella K, Altshuler D, Gabriel S, Daly M. 2010. The genome analysis toolkit: a MapReduce framework for analyzing next-generation DNA sequencing data. Genome Res. 20(9):1297–1303. 20644199 10.1101/gr.107524.110 PMC2928508

[msad199-B54] Mei J , Shao C, Yang R, Feng Y, Gao Y, Ding Y, Li J, Qian W. 2020. Introgression and pyramiding of genetic loci from wild *Brassica oleracea* into *B. napus* for improving Sclerotinia resistance of rapeseed. Theo Appl Genet. 133(4):1313–1319. 10.1007/s00122-020-03552-w 32008057

[msad199-B55] Meyer RS , DuVal AE, Jensen HR. 2012. Patterns and processes in crop domestication: an historical review and quantitative analysis of 203 global food crops. New Phytol. 196(1):29–48. 22889076 10.1111/j.1469-8137.2012.04253.x

[msad199-B56] Minh BQ , Schmidt HA, Chernomor O, Schrempf D, Woodhams MD, von Haeseler A, Lanfear R. 2020. IQ-TREE 2: new models and efficient methods for phylogenetic inference in the genomic era. Mol Biol Evol. 37(5):1530–1534. 32011700 10.1093/molbev/msaa015 PMC7182206

[msad199-B57] Monnahan P , Kolar F, Baduel P, Sailer C, Koch J, Horvath R, Laenen B, Schmickl R, Paajanen P, Sramkova G, et al 2019. Pervasive population genomic consequences of genome duplication in Arabidopsis arenosa. Nat Ecol Evol. 3(3):457–468. 30804518 10.1038/s41559-019-0807-4

[msad199-B58] Morales-Cruz A , Aguirre-Liguori JA, Zhou Y, Minio A, Riaz S, Walker AM, Cantu D, Gaut BS. 2021. Introgression among North American wild grapes (*Vitis*) fuels biotic and abiotic adaptation. Genome Biol. 22(1):254. 34479604 10.1186/s13059-021-02467-z PMC8414701

[msad199-B59] Nagaharu U . 1935. Genome analysis in *Brassica* with special reference to the experimental formation of *B. napus* and peculiar mode of fertilization. J Japan Bot. 7(7):389–452.

[msad199-B60] Nieto Feliner G , Casacuberta J, Wendel JF. 2020. Genomics of evolutionary novelty in hybrids and polyploids. Front Genet. 11:792. 32849797 10.3389/fgene.2020.00792 PMC7399645

[msad199-B61] Novikova PY , Brennan IG, Booker W, Mahony M, Doughty P, Lemmon AR, Moriarty Lemmon E, Roberts JD, Yant L, Van de Peer Y, et al 2020. Polyploidy breaks speciation barriers in Australian burrowing frogs *Neobatrachus*. PLoS Genet. 16(5):e1008769. 32392206 10.1371/journal.pgen.1008769 PMC7259803

[msad199-B62] Patterson N , Moorjani P, Luo Y, Mallick S, Rohland N, Zhan Y, Genschoreck T, Webster T, Reich D. 2012. Ancient admixture in human history. Genetics. 192(3):1065–1093. 22960212 10.1534/genetics.112.145037 PMC3522152

[msad199-B63] Perumal S , Koh CS, Jin L, Buchwaldt M, Higgins EE, Zheng C, Sankoff D, Robinson SJ, Kagale S, Navabi ZK, et al 2020. A high-contiguity *Brassica nigra* genome localizes active centromeres and defines the ancestral *Brassica* genome. Nat Plants. 6(8):929–941. 32782408 10.1038/s41477-020-0735-y PMC7419231

[msad199-B64] Petr M , Vernot B, Kelso J. 2019. . admixr—R package for reproducible analyses using ADMIXTOOLS. Bioinformatics. 35(17):3194–3195. 30668635 10.1093/bioinformatics/btz030 PMC6736366

[msad199-B65] Pickrell JK , Pritchard JK. 2012. Inference of population splits and mixtures from genome-wide allele frequency data. PLoS Genet. 8(11):e1002967. 23166502 10.1371/journal.pgen.1002967 PMC3499260

[msad199-B66] Purcell S , Neale B, Todd-Brown K, Thomas L, Ferreira MA, Bender D, Maller J, Sklar P, De Bakker PI, Daly MJ. 2007. PLINK: a tool set for whole-genome association and population-based linkage analyses. Am J Hum Genet. 81(3):559–575. 17701901 10.1086/519795 PMC1950838

[msad199-B67] Qi X , An H, Ragsdale AP, Hall TE, Gutenkunst RN, Chris Pires J, Barker MS. 2017. Genomic inferences of domestication events are corroborated by written records in *Brassica rapa*. Mol Ecol. 26(13):3373–3388. 28371014 10.1111/mec.14131

[msad199-B68] Qian W , Chen X, Fu D, Zou J, Meng J. 2005. Intersubgenomic heterosis in seed yield potential observed in a new type of *Brassica napus* introgressed with partial *Brassica rapa* genome. Theor Appl Genet. 110(7):1187–1194. 15806350 10.1007/s00122-005-1932-9

[msad199-B69] Raj A , Stephens M, Pritchard JK. 2014. fastSTRUCTURE: variational inference of population structure in large SNP data sets. Genetics. 197(2):573–589. 24700103 10.1534/genetics.114.164350 PMC4063916

[msad199-B70] Renny-Byfield S , Wendel JF. 2014. Doubling down on genomes: polyploidy and crop plants. Am J Bot. 101(10):1711–1725. 25090999 10.3732/ajb.1400119

[msad199-B71] Rieseberg LH , Carney SE. 1998. Plant hybridization. New Phytol. 140(4):599–624. 33862960 10.1046/j.1469-8137.1998.00315.x

[msad199-B72] Saban JM , Romero AJ, Ezard THG, Chapman MA, Sweigart A. 2023. Extensive crop–wild hybridization during Brassica evolution and selection during the domestication and diversification of Brassica crops. Genetics. 223(4):iyad027. 36810660 10.1093/genetics/iyad027 PMC10078912

[msad199-B73] Salman-Minkov A , Sabath N, Mayrose I. 2016. Whole-genome duplication as a key factor in crop domestication. Nat Plants. 2(8):16115. 27479829 10.1038/nplants.2016.115

[msad199-B74] Schmickl R , Yant L. 2021. Adaptive introgression: how polyploidy reshapes gene flow landscapes. New Phytol. 230(2):457–461. 33454987 10.1111/nph.17204

[msad199-B75] Schumer M , Xu C, Powell DL, Durvasula A, Skov L, Holland C, Blazier JC, Sankararaman S, Andolfatto P, Rosenthal GG, et al 2018. Natural selection interacts with recombination to shape the evolution of hybrid genomes. Science. 360(6389):656–660. 29674434 10.1126/science.aar3684 PMC6069607

[msad199-B76] Soltis DE , Albert VA, Leebens-Mack J, Bell CD, Paterson AH, Zheng C, Sankoff D, Depamphilis CW, Wall PK, Soltis PS. 2009. Polyploidy and angiosperm diversification. Am J Bot. 96(1):336–348. 21628192 10.3732/ajb.0800079

[msad199-B77] Soltis PS , Marchant DB, Van de Peer Y, Soltis DE. 2015. Polyploidy and genome evolution in plants. Curr Opin Genet Dev. 35:119–125. 26656231 10.1016/j.gde.2015.11.003

[msad199-B78] Soltis DE , Visger CJ, Soltis PS. 2014. The polyploidy revolution then…and now: Stebbins revisited. Am J Bot. 101(7):1057–1078. 25049267 10.3732/ajb.1400178

[msad199-B79] Song J-M , Guan Z, Hu J, Guo C, Yang Z, Wang S, Liu D, Wang B, Lu S, Zhou R, et al 2020. Eight high-quality genomes reveal pan-genome architecture and ecotype differentiation of *Brassica napus*. Nat Plants. 6(1):34–45. 31932676 10.1038/s41477-019-0577-7 PMC6965005

[msad199-B80] Stebbins GL . 1971. Chromosomal evolution in higher plants. London: Edward Arnold Ltd.

[msad199-B81] Suarez-Gonzalez A , Lexer C, Cronk QCB. 2018. Adaptive introgression: a plant perspective. Biol Lett. 14(3):20170688. 29540564 10.1098/rsbl.2017.0688 PMC5897607

[msad199-B82] Sun F , Fan G, Hu Q, Zhou Y, Guan MY, Tong C, Li J, Du D, Qi C, Jiang L, et al 2017. The high quality genome of *Brassica napus* cultivar ‘ZS11' Reveals the introgression history in semi-winter morphotype. Plant J. 92(3):452–468. 28849613 10.1111/tpj.13669

[msad199-B83] Szpiech ZA , Hernandez RD. 2014. . selscan: an efficient multithreaded program to perform EHH-based scans for positive selection. Mol Biol Evol. 31(10):2824–2827. 25015648 10.1093/molbev/msu211 PMC4166924

[msad199-B84] Tarasov A , Vilella AJ, Cuppen E, Nijman IJ, Prins P. 2015. Sambamba: fast processing of NGS alignment formats. Bioinformatics. 31(12):2032–2034. 25697820 10.1093/bioinformatics/btv098 PMC4765878

[msad199-B85] Tricou T , Tannier E, de Vienne DM. 2022. Ghost lineages highly influence the interpretation of introgression tests. Syst Biol. 71(5):1147–1158. 35169846 10.1093/sysbio/syac011 PMC9366450

[msad199-B86] Udall JA , Quijada PA, Polewicz H, Vogelzang R, Osborn TC. 2004. Phenotypic effects of introgressing Chinese winter and resynthesized *Brassica napus* L. germplasm into hybrid spring canola. Crop Sci. 44(6):1990–1996.

[msad199-B87] Udall JA , Wendel JF. 2006. Polyploidy and crop improvement. Crop Sci. 46:S3.

[msad199-B88] Van de Peer Y , Ashman T-L, Soltis PS, Soltis DE. 2020. Polyploidy: an evolutionary and ecological force in stressful times. Plant Cell. 33(1):11–26. 10.1093/plcell/koaa015 PMC8136868 33751096

[msad199-B89] Van de Peer Y , Mizrachi E, Marchal K. 2017. The evolutionary significance of polyploidy. Nat Rev Genet. 18(7):411–424. 28502977 10.1038/nrg.2017.26

[msad199-B90] Wang L , Beissinger TM, Lorant A, Ross-Ibarra C, Ross-Ibarra J, Hufford MB. 2017. The interplay of demography and selection during maize domestication and expansion. Genome Biol. 18(1):215. 29132403 10.1186/s13059-017-1346-4 PMC5683586

[msad199-B91] Wang Y , Schuck S, Wu J, Yang P, Döring A-C, Zeier J, Tsuda K. 2018. A MPK3/6-WRKY33-ALD1-pipecolic acid regulatory loop contributes to systemic acquired resistance. Plant Cell. 30(10):2480–2494. 30228125 10.1105/tpc.18.00547 PMC6241261

[msad199-B92] Whitney KD , Randell RA, Rieseberg LH. 2010. Adaptive introgression of abiotic tolerance traits in the sunflower *Helianthus annuus*. New Phytol. 187(1):230–239. 20345635 10.1111/j.1469-8137.2010.03234.x

[msad199-B93] Wu T , Hu E, Xu S, Chen M, Guo P, Dai Z, Feng T, Zhou L, Tang W, Zhan L, et al 2021. Clusterprofiler 4.0: a universal enrichment tool for interpreting omics data. Innovation. 2(3):100141. 34557778 10.1016/j.xinn.2021.100141 PMC8454663

[msad199-B94] Wu D , Liang Z, Yan T, Xu Y, Xuan L, Tang J, Zhou G, Lohwasser U, Hua S, Wang H, et al 2019. Whole-genome resequencing of a worldwide collection of rapeseed accessions reveals the genetic basis of ecotype divergence. Mol Plant. 12(1):30–43. 30472326 10.1016/j.molp.2018.11.007

[msad199-B95] Yang J , Liu D, Wang X, Ji C, Cheng F, Liu B, Hu Z, Chen S, Pental D, Ju Y, et al 2016. The genome sequence of allopolyploid *Brassica juncea* and analysis of differential homoeolog gene expression influencing selection. Nat Genet. 48:1225. 27595476 10.1038/ng.3657

[msad199-B96] Yang Y , Shen Y, Li S, Ge X, Li Z. 2017. High density linkage map construction and QTL detection for three silique-related traits in *Orychophragmus violaceus* derived *Brassica napus* population. Front Plant Sci. 8:1512. 28932230 10.3389/fpls.2017.01512 PMC5592274

[msad199-B97] Yang J , Zhang C, Zhao N, Zhang L, Hu Z, Chen S, Zhang M. 2018. Chinese root-type mustard provides phylogenomic insights into the evolution of the multi-use diversified allopolyploid *Brassica juncea*. Mol Plant. 11(3):512–514. 29183772 10.1016/j.molp.2017.11.007

[msad199-B98] Yu G . 2020. Using ggtree to visualize data on tree-like structures. Curr Protoc Bioinformatics. 69(1):e96. 32162851 10.1002/cpbi.96

[msad199-B99] Zhang L , Cai X, Wu J, Liu M, Grob S, Cheng F, Liang J, Cai C, Liu Z, Liu B, et al 2018. Improved *Brassica rapa* reference genome by single-molecule sequencing and chromosome conformation capture technologies. Hortic Res. 5(1):50. 30131865 10.1038/s41438-018-0071-9 PMC6092429

[msad199-B100] Zhang C , Dong S-S, Xu J-Y, He W-M, Yang T-L. 2018. PopLDdecay: a fast and effective tool for linkage disequilibrium decay analysis based on variant call format files. Bioinformatics. 35(10):1786–1788. 10.1093/bioinformatics/bty875 30321304

[msad199-B101] Zhang L , Li X, Chang L, Wang T, Liang J, Lin R, Wu J, Wang X. 2022. Expanding the genetic variation of *Brassica juncea* by introgression of the *Brassica rapa* genome. Hortic Res. 9:uhab054. 35043197 10.1093/hr/uhab054 PMC8883073

[msad199-B102] Zhao Y , Zhang R, Jiang KW, Qi J, Hu Y, Guo J, Zhu R, Zhang T, Egan AN, Yi TS, et al 2021. Nuclear phylotranscriptomics and phylogenomics support numerous polyploidization events and hypotheses for the evolution of rhizobial nitrogen-fixing symbiosis in Fabaceae. Mol Plant. 14(5):748–773. 33631421 10.1016/j.molp.2021.02.006

[msad199-B103] Zhou Y , Zhao X, Li Y, Xu J, Bi A, Kang L, Xu D, Chen H, Wang Y, Wang Y-G, et al 2020. *Triticum* population sequencing provides insights into wheat adaptation. Nat Genet. 52(12):1412–1422. 33106631 10.1038/s41588-020-00722-w

[msad199-B104] Zohren J , Wang N, Kardailsky I, Borrell JS, Joecker A, Nichols RA, Buggs RJ. 2016. Unidirectional diploid-tetraploid introgression among British birch trees with shifting ranges shown by restriction site-associated markers. Mol Ecol. 25(11):2413–2426. 27065091 10.1111/mec.13644 PMC4999052

[msad199-B105] Zou J , Mao L, Qiu J, Wang M, Jia L, Wu D, He Z, Chen M, Shen Y, Shen E, et al 2019. Genome-wide selection footprints and deleterious variations in young Asian allotetraploid rapeseed. Plant Biotech J. 17(10):1998–2010. doi:10.1111/pbi.13115 PMC6737024 30947395

