# Supplementary material for: Interploidy Introgression Shaped Adaptation during the Origin and Domestication History of Brassica napus
Source: Mol Biol Evol. 2023 Sep 14;40(9):msad199. doi: 10.1093/molbev/msad199 (PMC10504873; doi:10.1093/molbev/msad199)
Supplement: msad199_Supplementary_Data [file msad199_supplementary_data.zip › 3.SupplementaryFigures.pdf]

Supplementary Figures for

Interploidy introgression shaped adaptation during the origin and domestication history of *Brassica napus*

Tianpeng Wang[1,2,3,4], Aalt D.J. van Dijk[4], Johan Bucher[3], Jianli Liang[1,2], Jian Wu[1,2], Guusje Bonnema[2,3*], Xiaowu Wang[1,2*]

[1]State Key Laboratory of Vegetable Biobreeding, Institute of Vegetables and Flowers, Chinese Academy of Agricultural Sciences, Beijing, China

[2]Sino-Dutch Joint Laboratory of Horticultural Genomics, Institute of Vegetables and Flowers, Chinese Academy of Agricultural Sciences, Beijing, China.

[3]Plant Breeding, Wageningen University and Research, Wageningen, The Netherlands

[4]Bioinformatics Group, Wageningen University and Research, Wageningen, The Netherlands

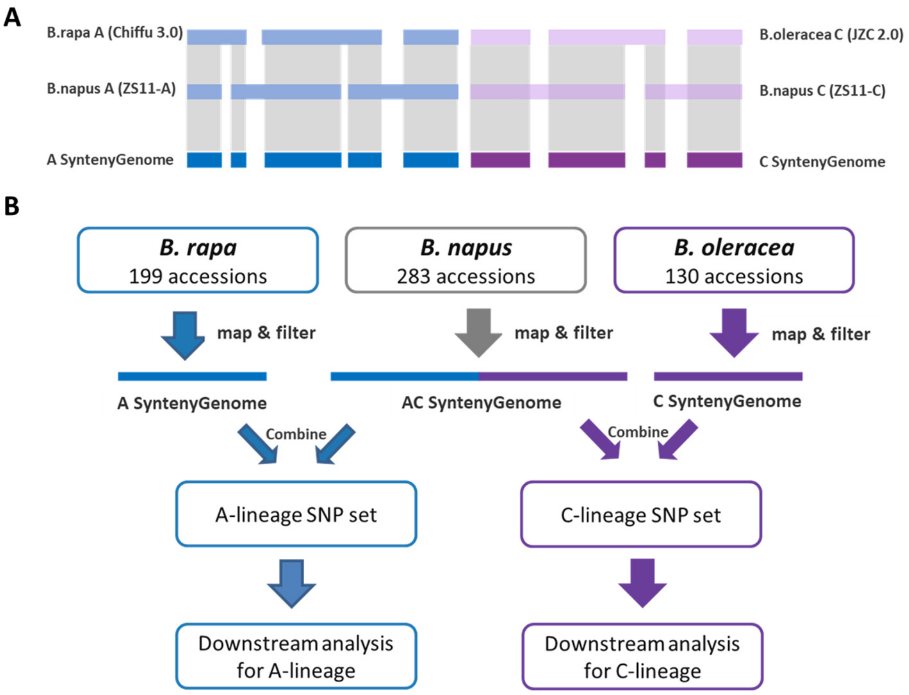

Supplementary Figure S1. The workflow of cross-ploidy variation discovery and downstream analysis.

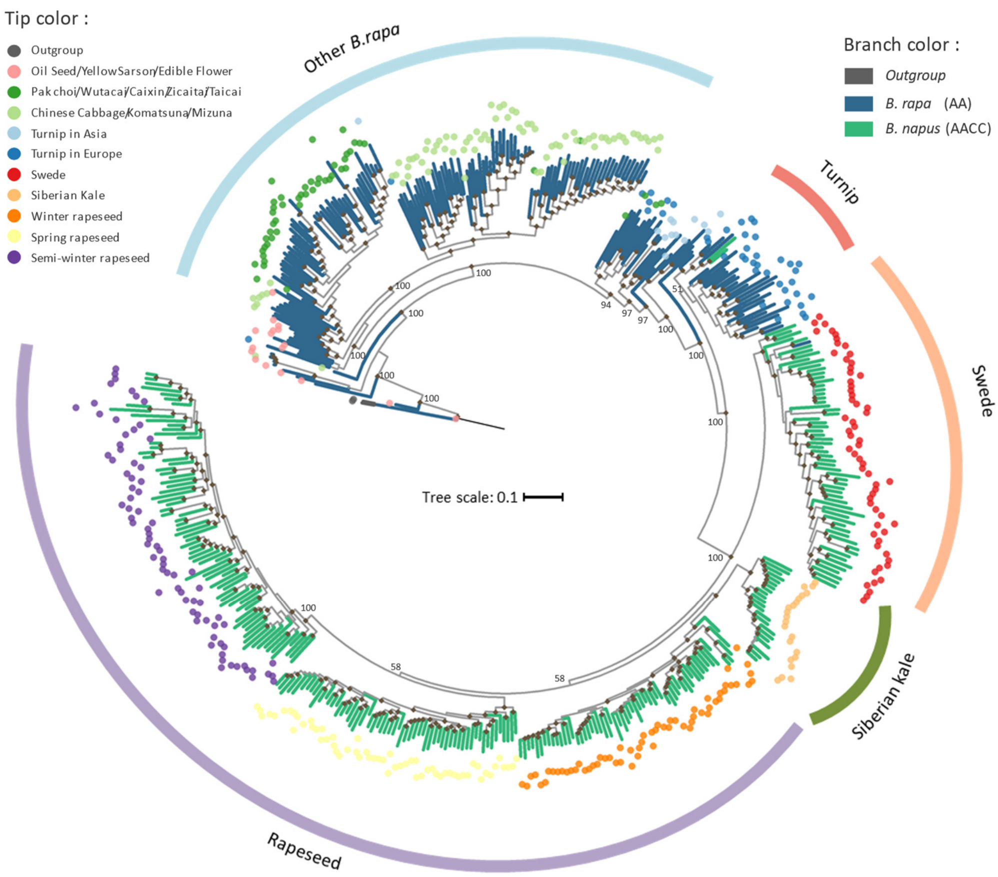

Supplementary Figure S2. Phylogenetic tree of A lineage based on 200k randomly chosen SNPs. Branch colors of phylogeny represent subspecies/morphotypes. Branches with reliable bootstrap value (>70) are labeled with a black rectangle at corresponding nodes.

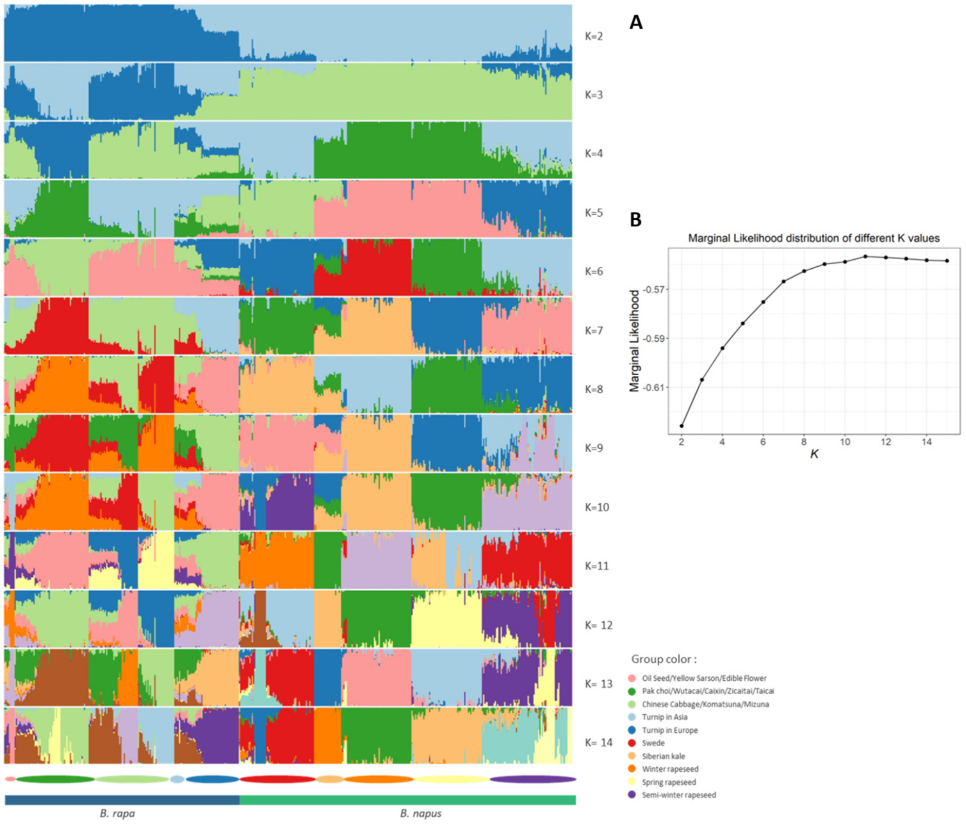

Supplementary Figure S3. (*A*) Population structure of A lineage by fastSTRUCTURE analysis for the K ranged from 2 to 14. Each colored bar represents one individual, and colored segments represent proportions of ancestral components. (*B*) Marginal Likelihood value for different K values in fastSTRUCTURE.

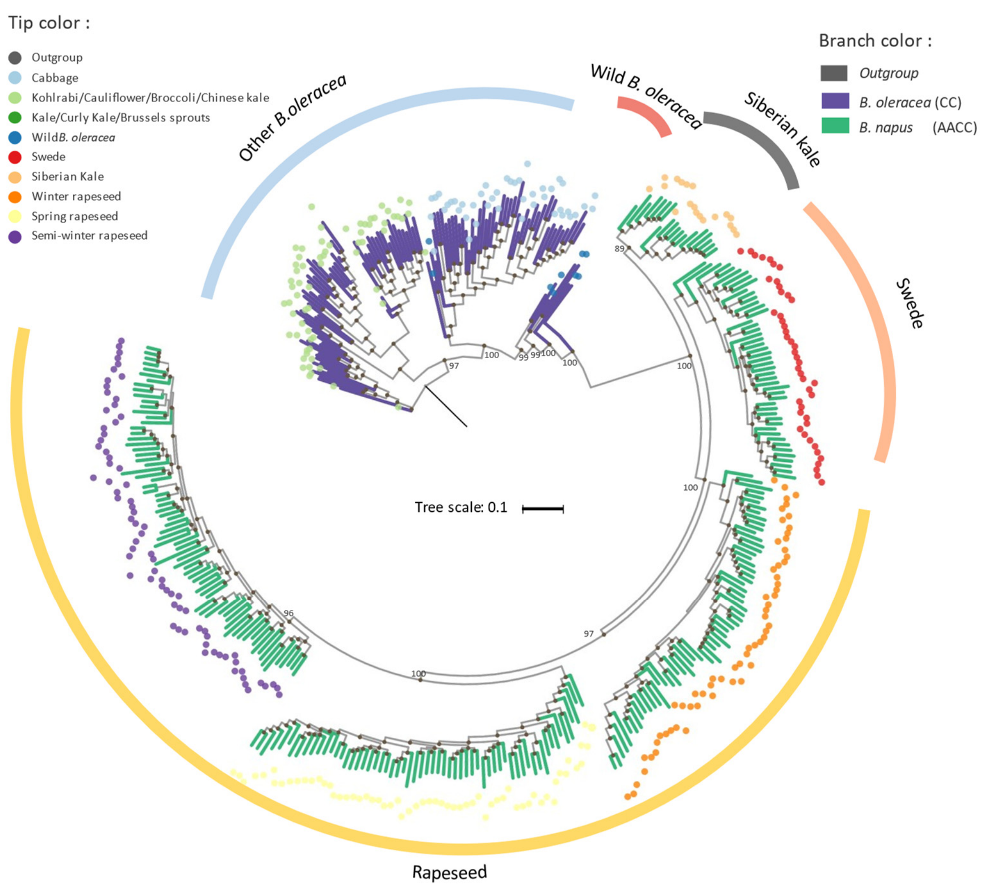

Supplementary Figure S4. Phylogenetic tree of C lineage based on 200k randomly chosen SNPs. Branch colors of phylogeny represent subspecies/morphotypes. Branches with reliable bootstrap value (>70) are labeled with a black rectangle at corresponding nodes.

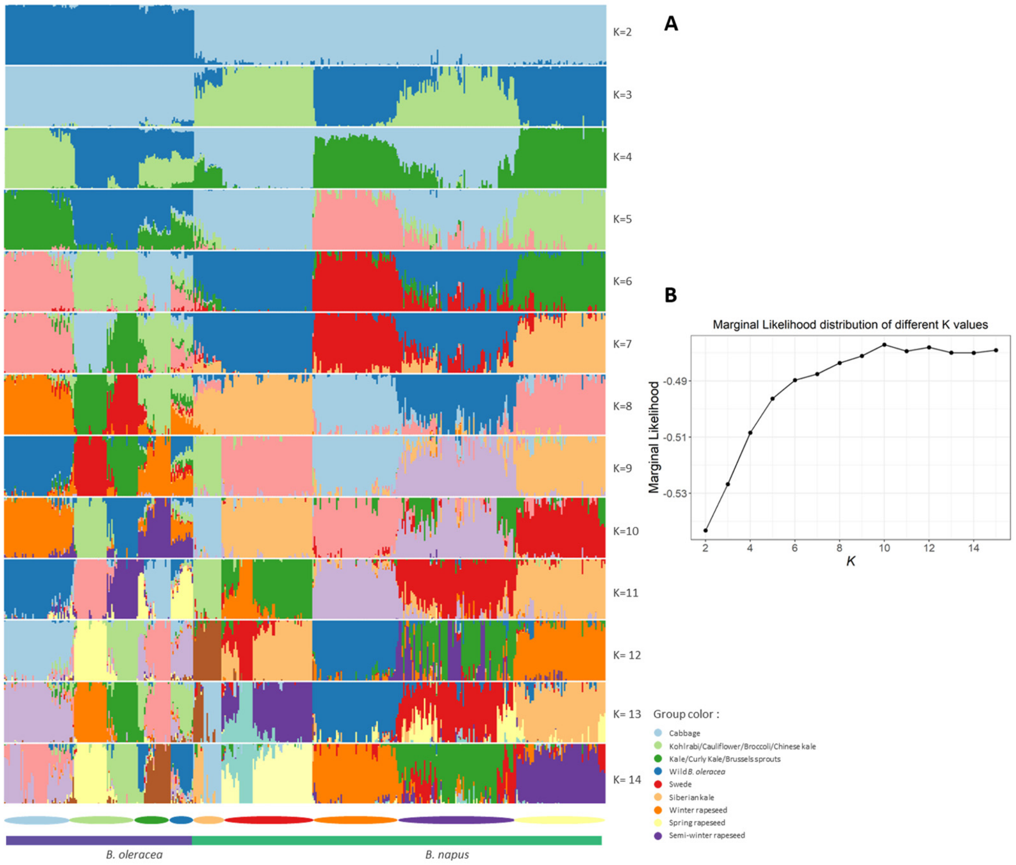

Supplementary Figure S5. (*A*) Population structure of C lineage by fastSTRUCTURE analysis for the K ranged from 2 to 14. Each colored bar represents one individual, and colored segments represent proportions of ancestral components. (*B*) Marginal Likelihood value for different K values in fastSTRUCTURE.

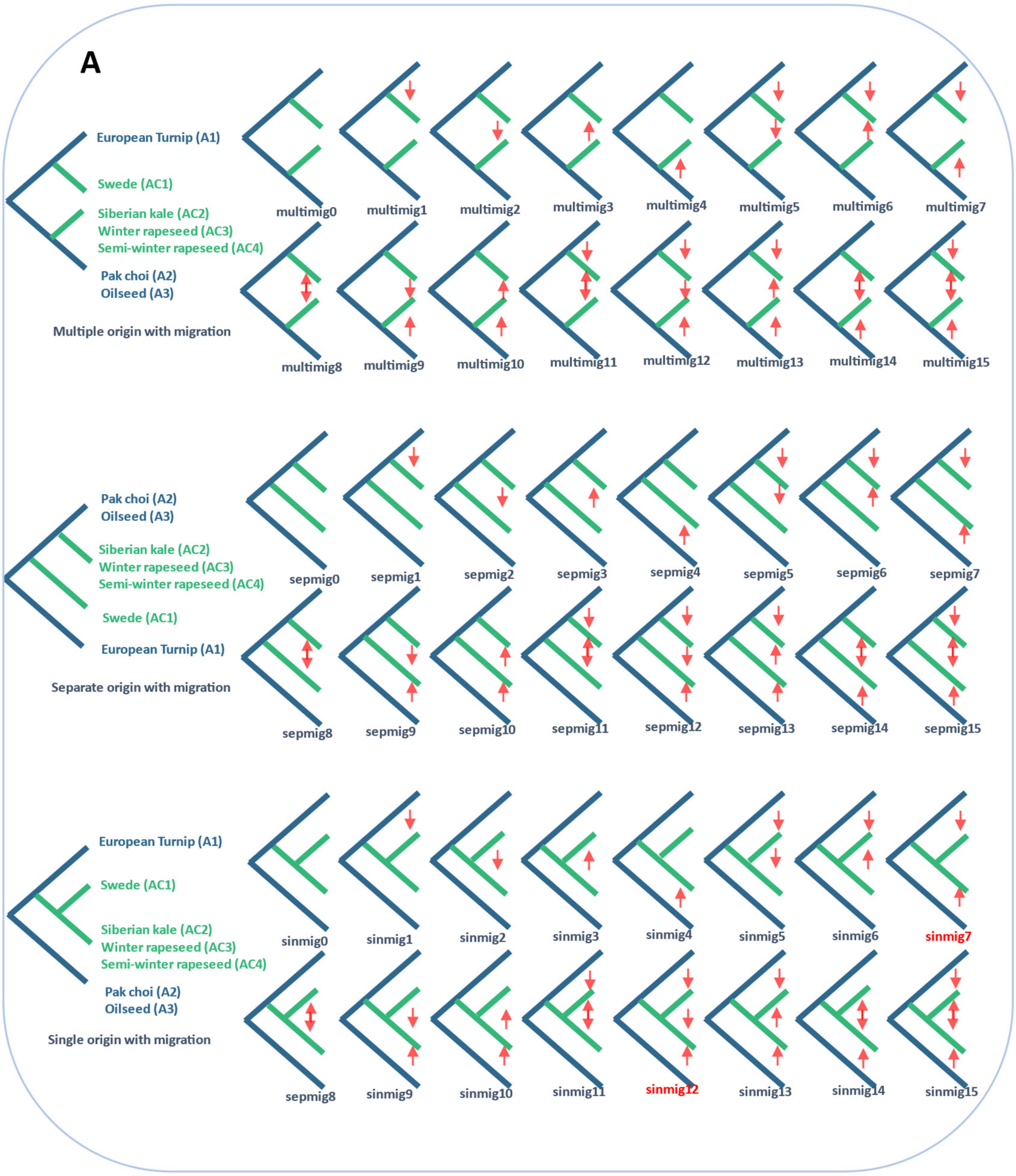
A
European Turnip (A1)
Swede (AC1)
Siberian kale (AC2)
Winter rapeseed (AC3)
Semi-winter rapeseed (AC4)
Pak choi (A2)
Oilseed (A3)
Multiple origin with migration
multimig0
multimig1
multimig2
multimig3
multimig4
multimig5
multimig6
multimig7
multimig8
multimig9
multimig10
multimig11
multimig12
multimig13
multimig14
multimig15
Pak choi (A2)
Oilseed (A3)
Siberian kale (AC2)
Winter rapeseed (AC3)
Semi-winter rapeseed (AC4)
Swede (AC1)
European Turnip (A1)
Separate origin with migration
sepmig0
sepmig1
sepmig2
sepmig3
sepmig4
sepmig5
sepmig6
sepmig7
sepmig8
sepmig9
sepmig10
sepmig11
sepmig12
sepmig13
sepmig14
sepmig15
European Turnip (A1)
Swede (AC1)
Siberian kale (AC2)
Winter rapeseed (AC3)
Semi-winter rapeseed (AC4)
Pak choi (A2)
Oilseed (A3)
Single origin with migration
sinmig0
sinmig1
sinmig2
sinmig3
sinmig4
sinmig5
sinmig6
sinmig7
sepmig8
sinmig9
sinmig10
sinmig11
sinmig12
sinmig13
sinmig14
sinmig15

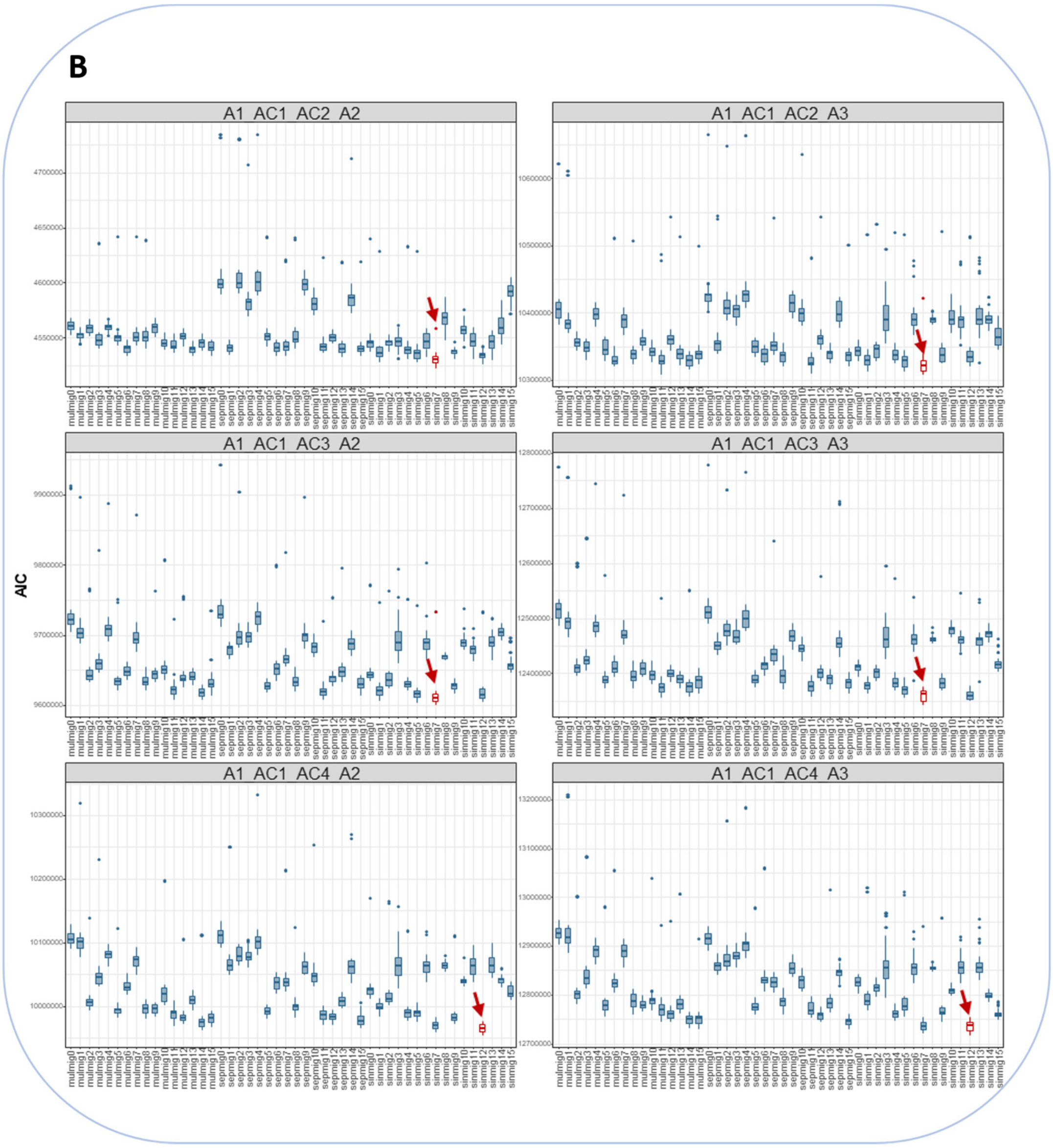

Supplementary Figure S6. Comparison of fastsimcoal2 demographic models and their AIC values for A lineage approximating the origin of *B. napus*. (***A***) Schematic diagram of alternative demographic models used in fastsimcoal2. Different models were employed to investigate the origin of the A subgenome in *B. napus*. Three major scenarios consisting of 48 models were considered: 1) multiple origin from distinct diploid *B. rapa* populations (mulmig), 2) separate polyploid origin from the same diploid progenitors (sepmig), 3) single origin from the same diploid population (sinmig). (***B***) Comparison of Akaike information criteria (AIC) across models. Each scenario was simulated by 30

independent runs of fastsimcoal2 for various combination of population quartets. The red arrow highlights the model with the consistently lowest AIC values within a particular population quartet, which is preferred by fastsimcoal2 (ΔAIC > 2).

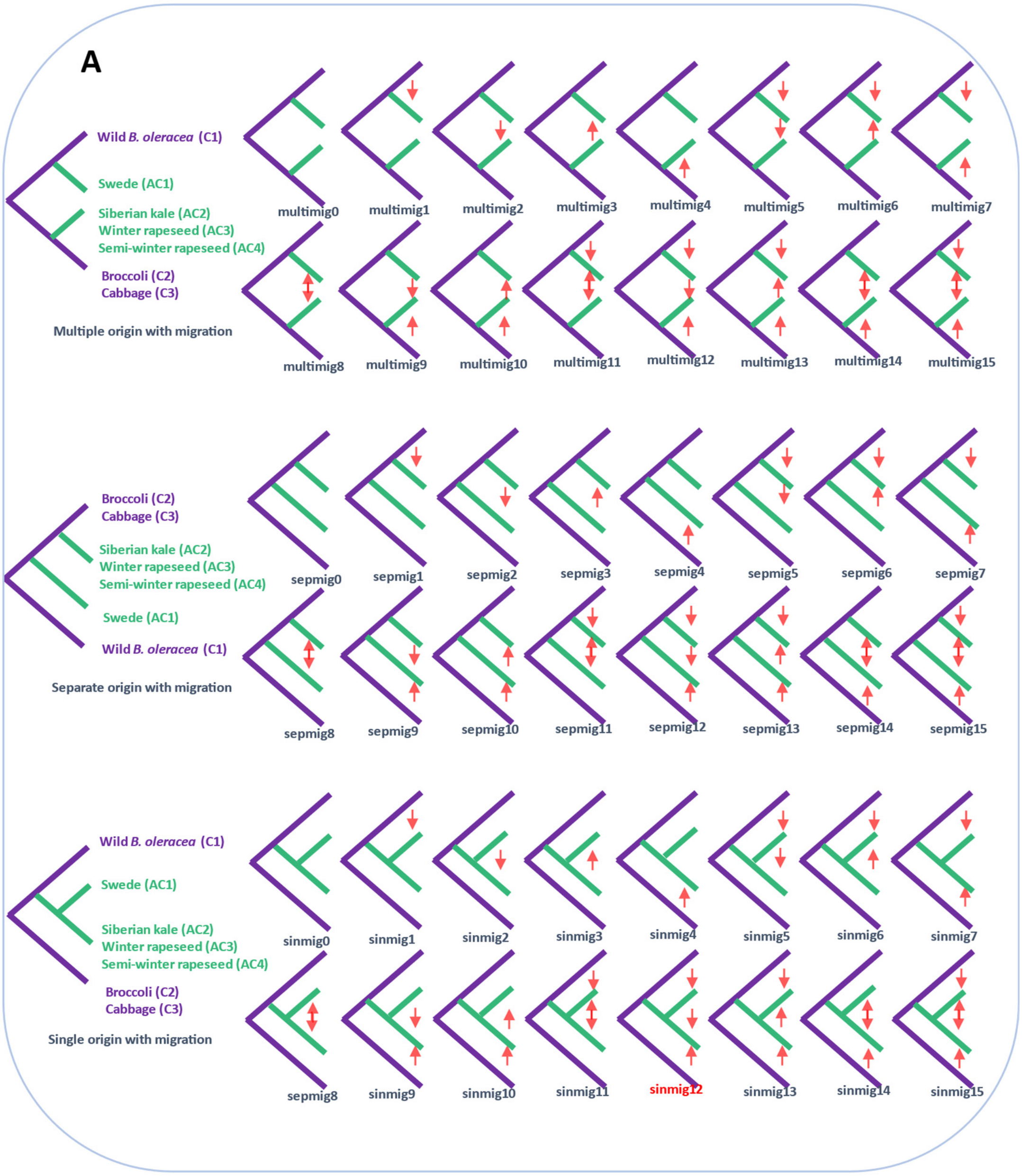
A
Wild B. oleracea (C1)
Swede (AC1)
Siberian kale (AC2)
Winter rapeseed (AC3)
Semi-winter rapeseed (AC4)
Broccoli (C2)
Cabbage (C3)
Multiple origin with migration
multimig0
multimig1
multimig2
multimig3
multimig4
multimig5
multimig6
multimig7
multimig8
multimig9
multimig10
multimig11
multimig12
multimig13
multimig14
multimig15
Broccoli (C2)
Cabbage (C3)
Siberian kale (AC2)
Winter rapeseed (AC3)
Semi-winter rapeseed (AC4)
Swede (AC1)
Wild B. oleracea (C1)
Separate origin with migration
sepmig0
sepmig1
sepmig2
sepmig3
sepmig4
sepmig5
sepmig6
sepmig7
sepmig8
sepmig9
sepmig10
sepmig11
sepmig12
sepmig13
sepmig14
sepmig15
Wild B. oleracea (C1)
Swede (AC1)
Siberian kale (AC2)
Winter rapeseed (AC3)
Semi-winter rapeseed (AC4)
Broccoli (C2)
Cabbage (C3)
Single origin with migration
sinmig0
sinmig1
sinmig2
sinmig3
sinmig4
sinmig5
sinmig6
sinmig7
sepmig8
sinmig9
sinmig10
sinmig11
sinmig12
sinmig13
sinmig14
sinmig15

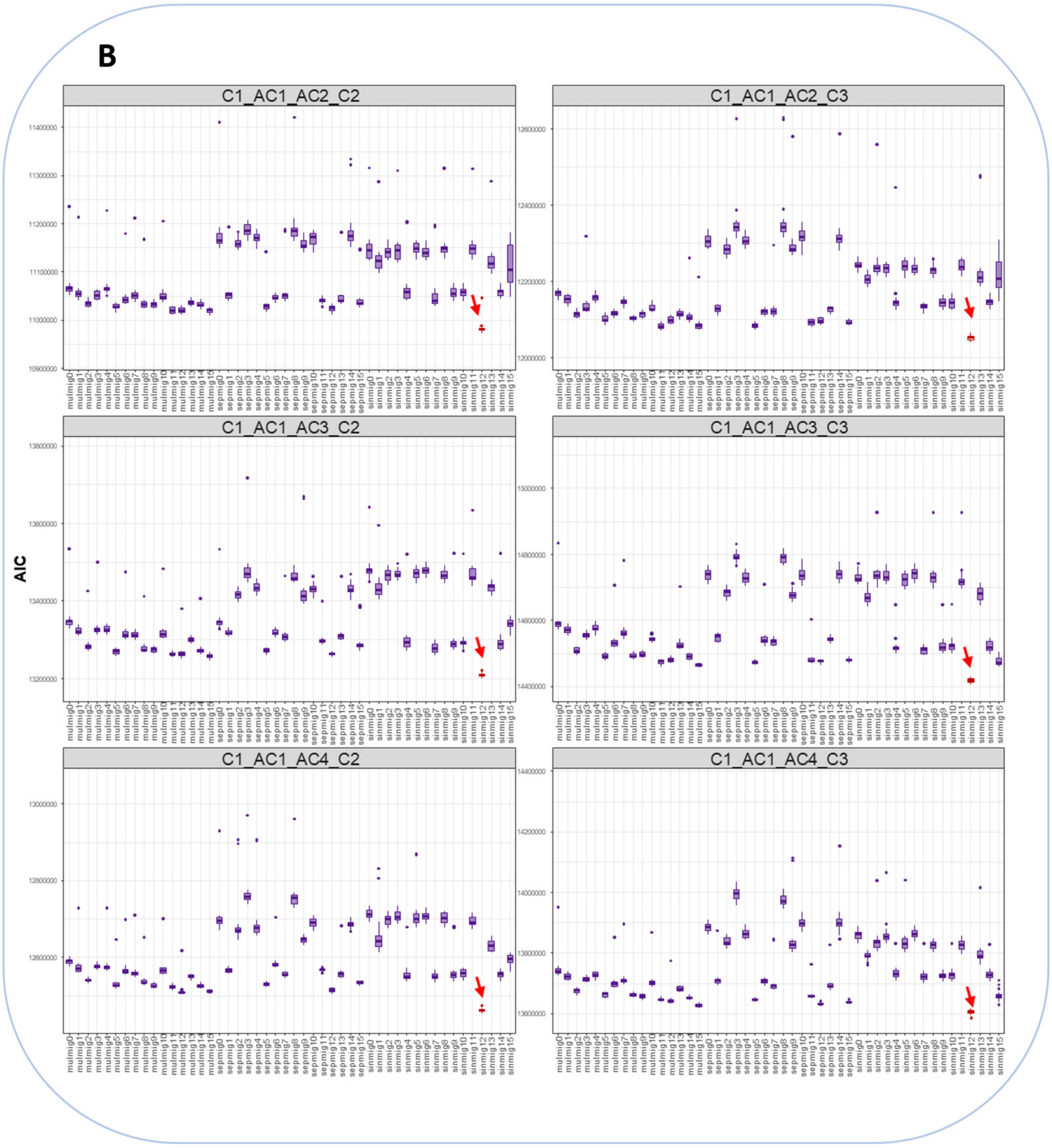

Supplementary Figure S7. Comparison of fastsimcoal2 demographic models and their AIC values for C lineage approximating the origin of *B. napus*. (*A*) Schematic diagram of alternative demographic models used in fastsimcoal2. Different models were employed to investigate the origin of the C subgenome in *B. napus*. Three major scenarios consisting of 48 models were considered 1) multiple origin from distinct diploid *B. oleracea* populations (mulmig), 2) separate polyploid origin from the same diploid progenitors (sepmig), 3) single origin from the same diploid population (sinmig). (*B*) Comparison of Akaike information criteria (AIC) across models. Each scenario was simulated by 30

independent runs of fastsimcoal2 for various combination of population quartets. The red arrow highlights the model with the consistently lowest AIC values within a particular population quartet, which is preferred by fastsimcoal2 (ΔAIC > 2).

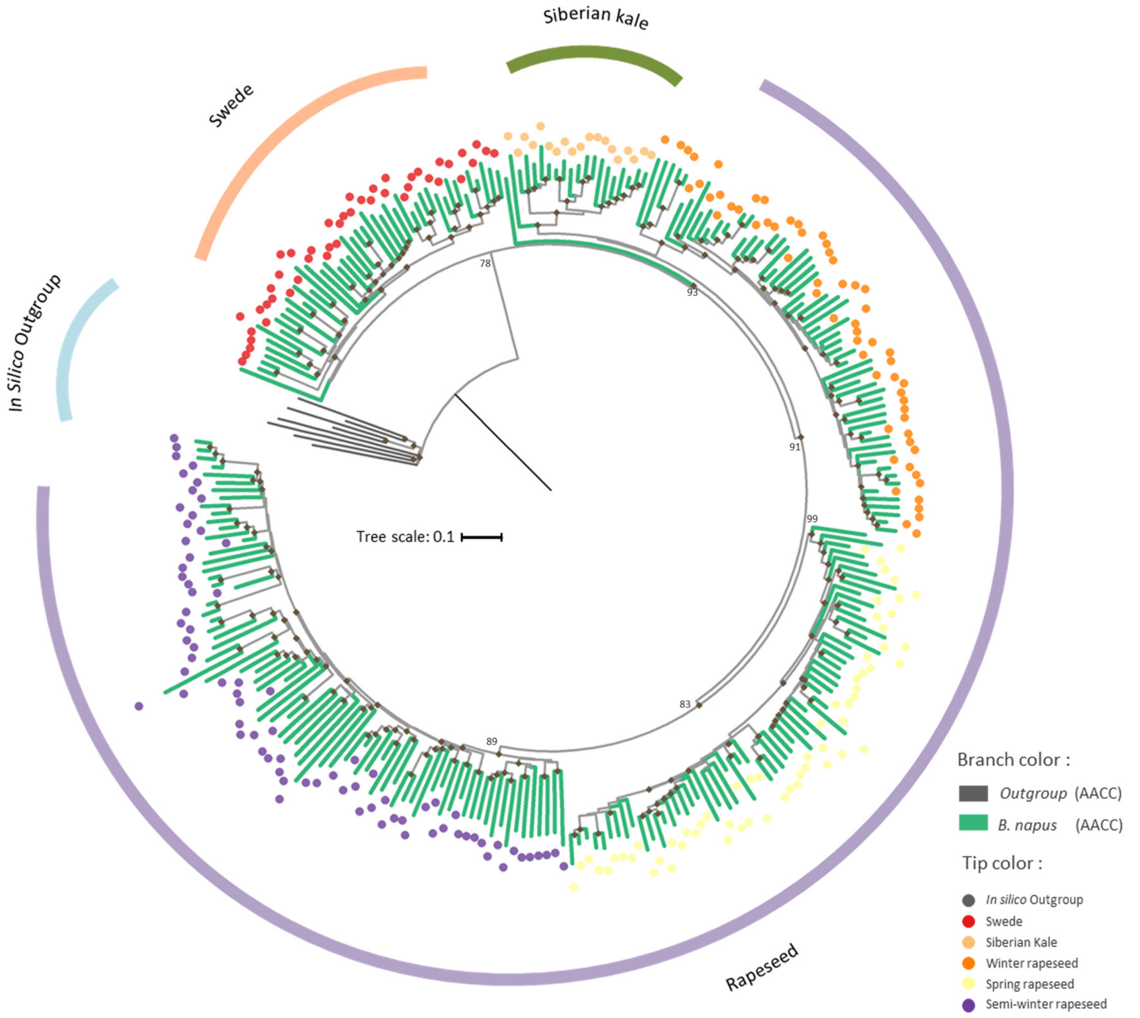

Supplementary Figure S8. Phylogenetic tree of *B. napus* based on a combination of 150,000 SNPs from each subgenome. Outgroup represents in silico ancestry of *B. napus* which is formed by combination of 8 randomly selected European turnip (*B. rapa*) and Wild *B. oleracea* accessions. Branch colors of phylogeny represent subspecies/morphotypes. Branches with reliable bootstrap value (>80) are labeled with a black rectangle at corresponding nodes.

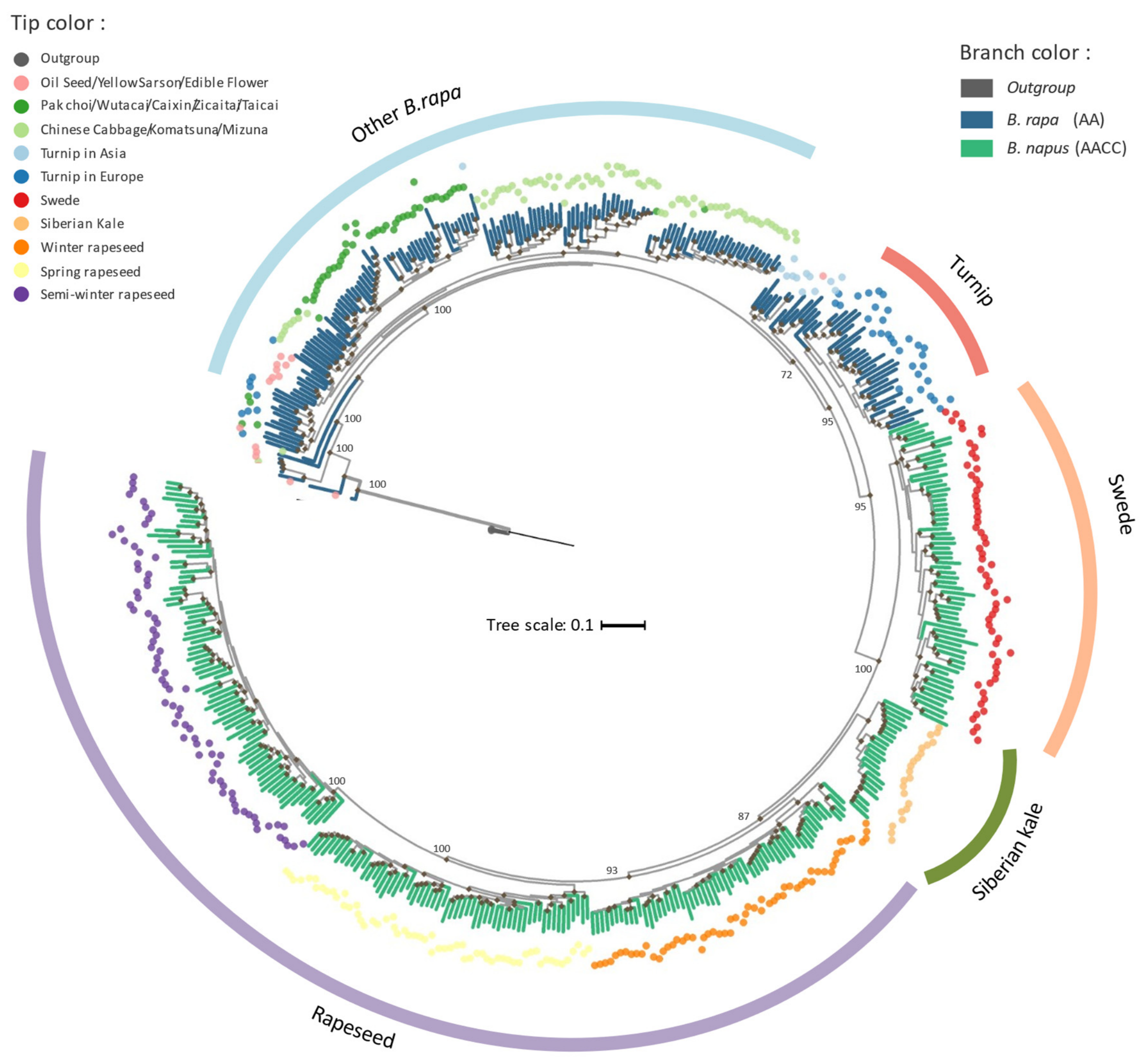

Supplementary Figure S9. Phylogenetic tree of A lineage removing SNPs from introgressed regions of swede group. Branch colors of phylogeny represent subspecies/morphotypes. Branches with reliable bootstrap value (>80) are labeled with a black rectangle at corresponding nodes.

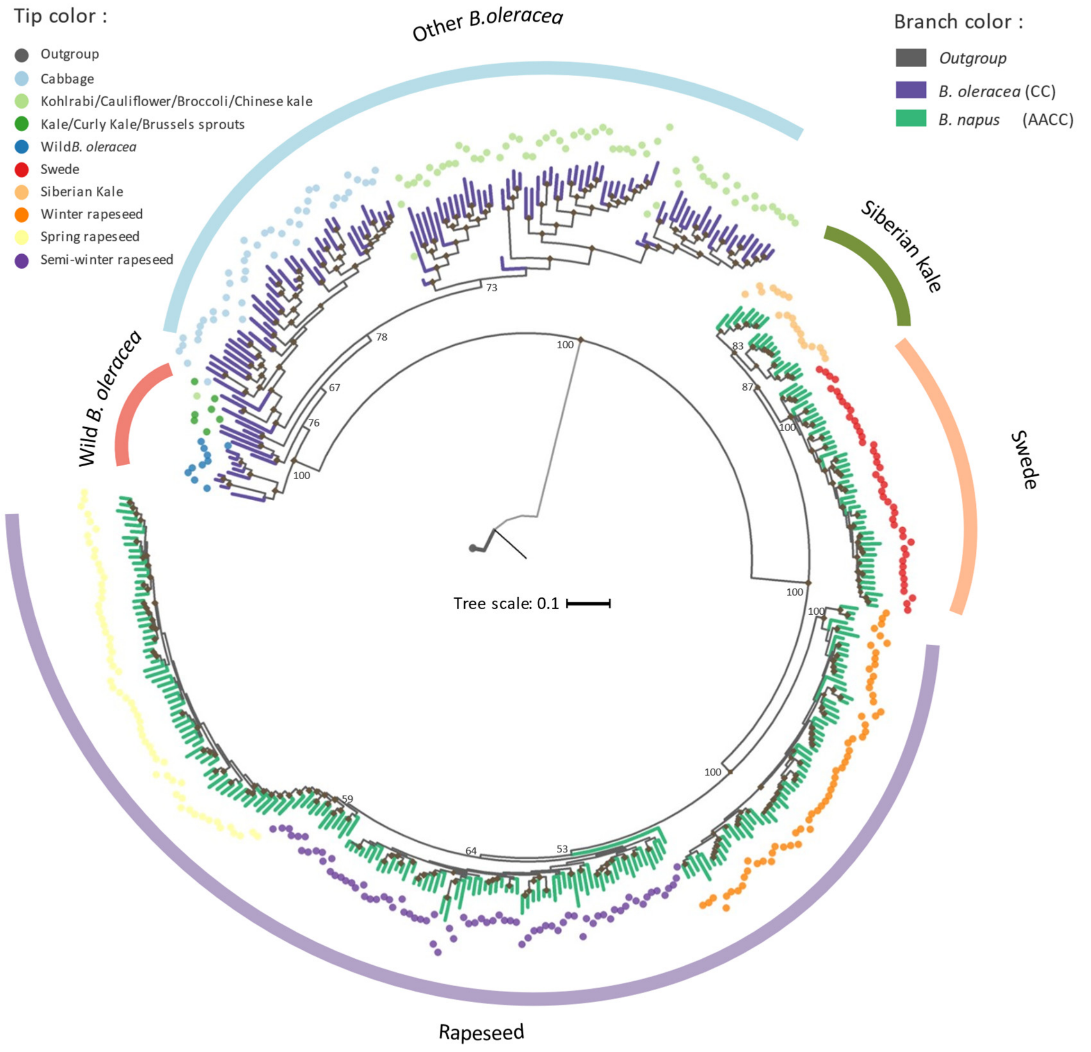

Supplementary Figure S10. Phylogenetic tree of C lineage removing SNPs from introgressed regions of Siberian kale group. Branch colors of phylogeny represent subspecies/morphotypes. Branches with reliable bootstrap value (>70) are labeled with a black rectangle at corresponding nodes.

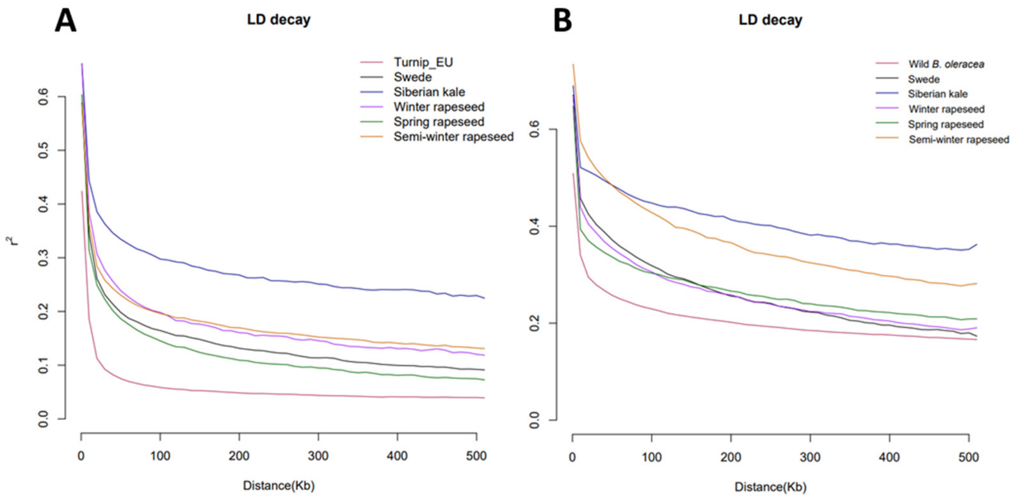

Supplementary Figure S11. Decay of linkage disequilibrium (LD) in different morphotypes measured by r^2. (*A*) LD decay of European turnip (*B. rapa*) and A subgenome of different populations from *B. napus.* (*B*) LD decay of Wild relatives of *B. oleracea* and C subgenome of different populations from *B. napus*.

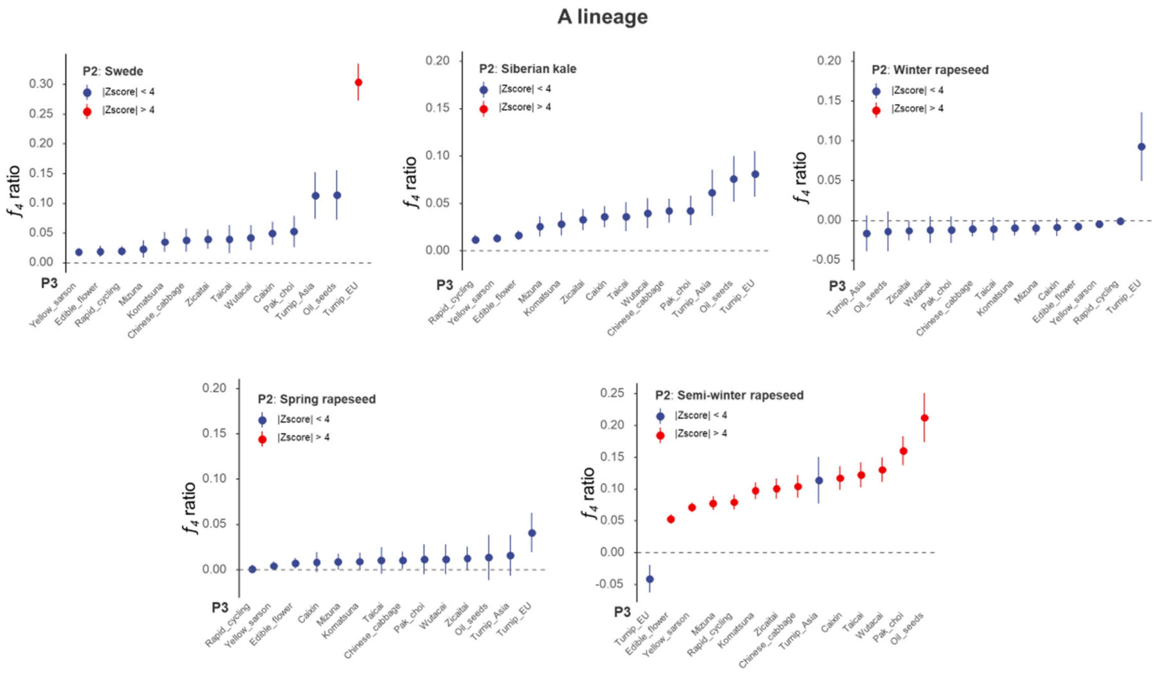

Supplementary Figure S12. Interploidy introgression in A lineage detected by *f_4*-ratio statistics. f_4-ratio statistics to test the proportion of interploidy introgression in specific groups. Filled red circles indicate a significant value ($|Z| > 4$); blue circles, $|Z| < 4$. The top and bottom whiskers correspond to 1 s.e. calculated across A subgenomes using a weighted blocked jackknife.

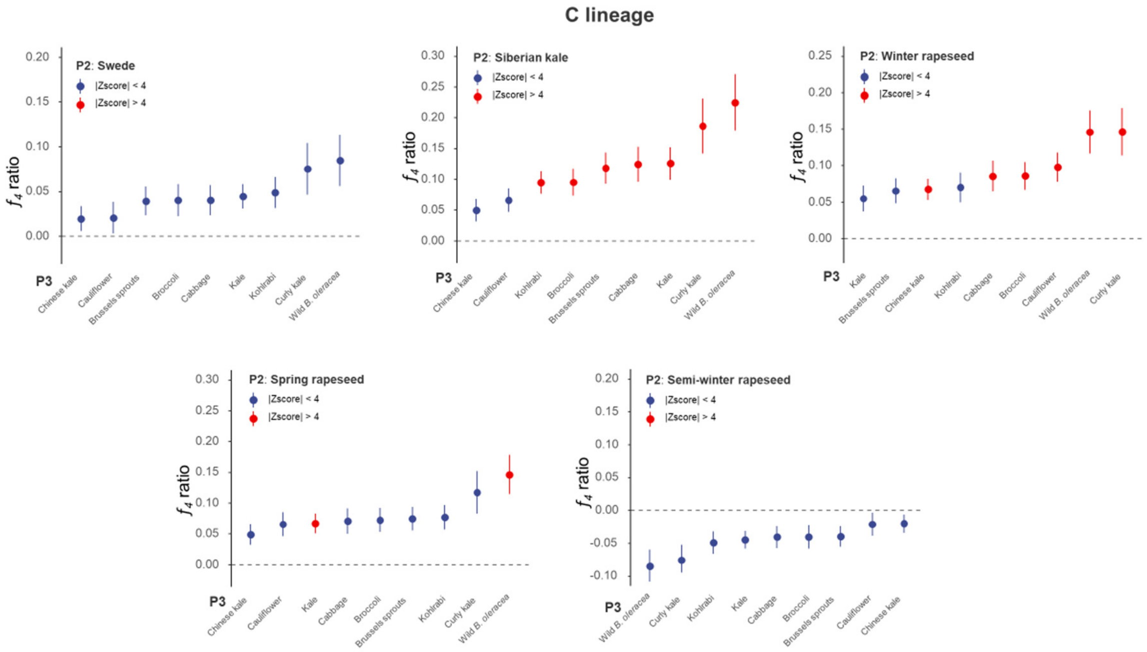

Supplementary Figure S13. Interploidy introgression in C lineage detected by f4-ratio statistics. f_4-ratio statistics to test the proportion of interploidy introgression in specific groups. Filled red circles indicate a significant value (|*Z*| > 4); blue circles, |*Z*| < 4. The top and bottom whiskers correspond to 1 s.e. calculated across C subgenomes using a weighted blocked jackknife.

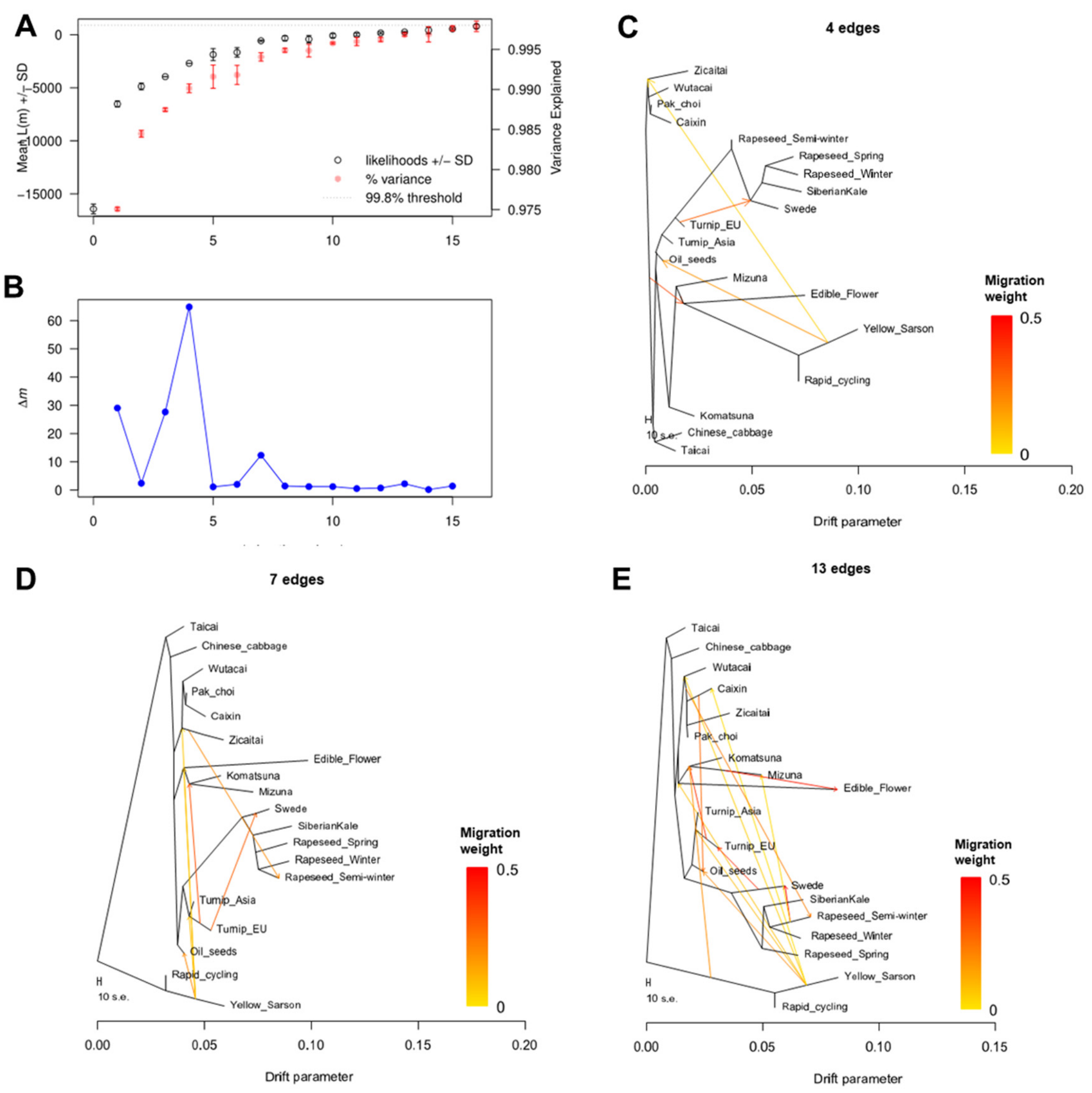

Supplementary Figure S14. Gene flow events among different populations in A lineage estimated by Treemix and OptM. (*A*) The mean and standard deviation (SD) across 3 iterations for the composite likelihood (left axis) and proportion of variance (right axis) explained by models with 1-15 edges. (*B*) Distribution of deltaM statistic based on the second order rate of change in the likelihood with standard deviation considered. (*C-E*) Three models with different migration events that achieved >99.5% variance explained and the high deltaM values.

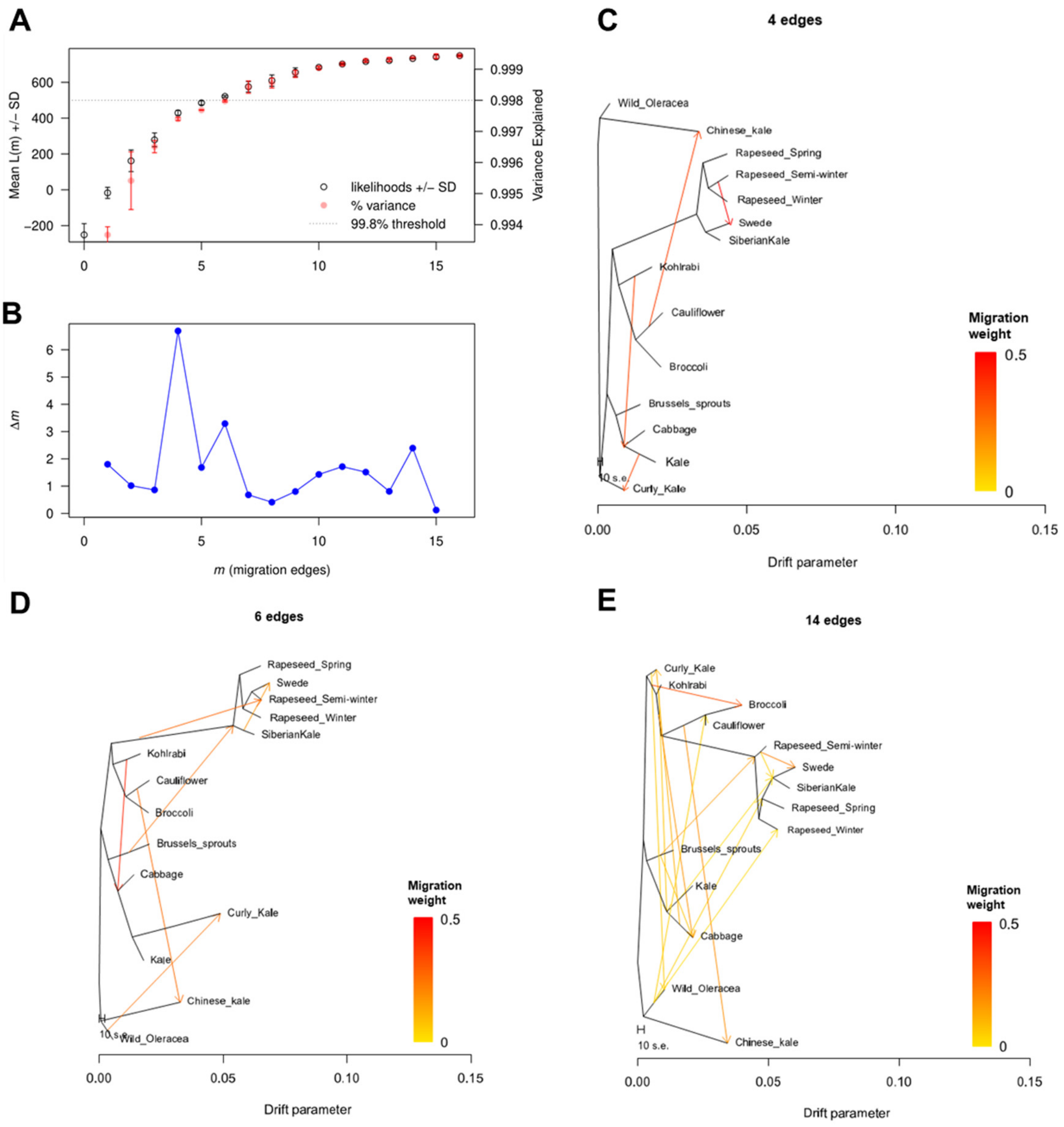

Supplementary Figure S15. Gene flow events among different populations in C lineage estimated by Treemix and OptM. (*A*) The mean and standard deviation (SD) across 3 iterations for the composite likelihood (left axis) and proportion of variance (right axis) explained by models with 1-15 edges. (*B*) Distribution of deltaM statistic based on the second order rate of change in the likelihood with standard deviation considered. (*C*-*E*) Three models with different migration events that achieved >99.5% variance explained and the high deltaM values.

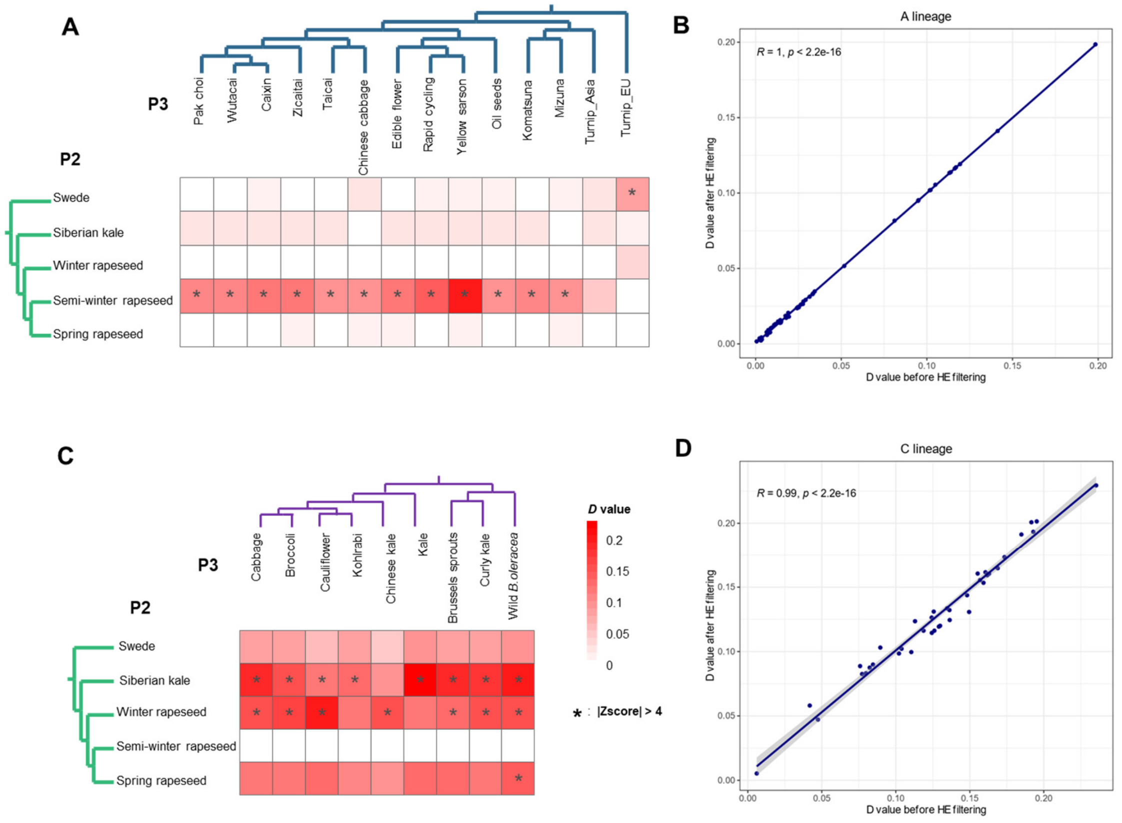

Supplementary Figure S16. Examination of potential impact of homoeologous exchange (HE) on inferring interploidy introgression. (*A, C*) Heatmaps indicate maximum pairwise Patterson's D statistics measurements between pairs of morphotypes across all combinations in A and C lineages after filtering HE-influenced SNPs. Asterisks indicate a significant value (|Z| > 4). (*B, D*) Correlation between the D statistics value with and without exclusion of HE regions (Pearson's correlation).

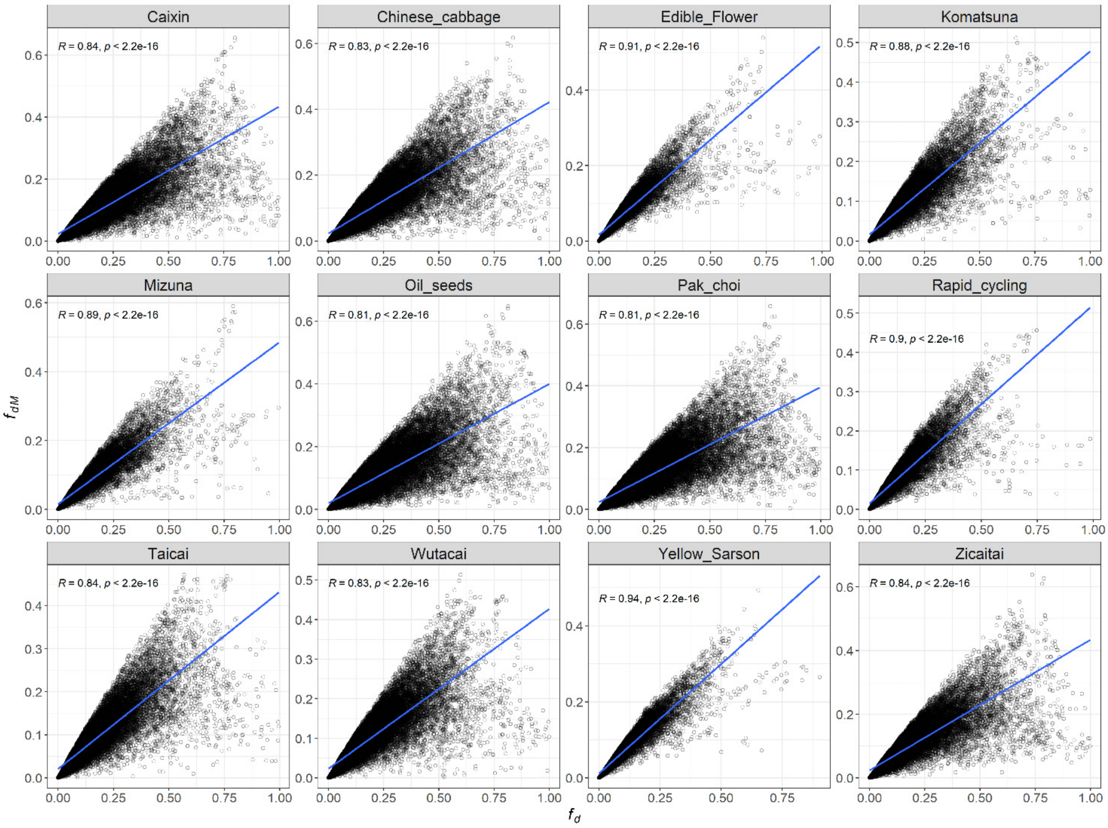

Supplementary Figure S17. Scatterplot showing correlations between f_d and f_{dM} statistics across windows among the 12 trios.

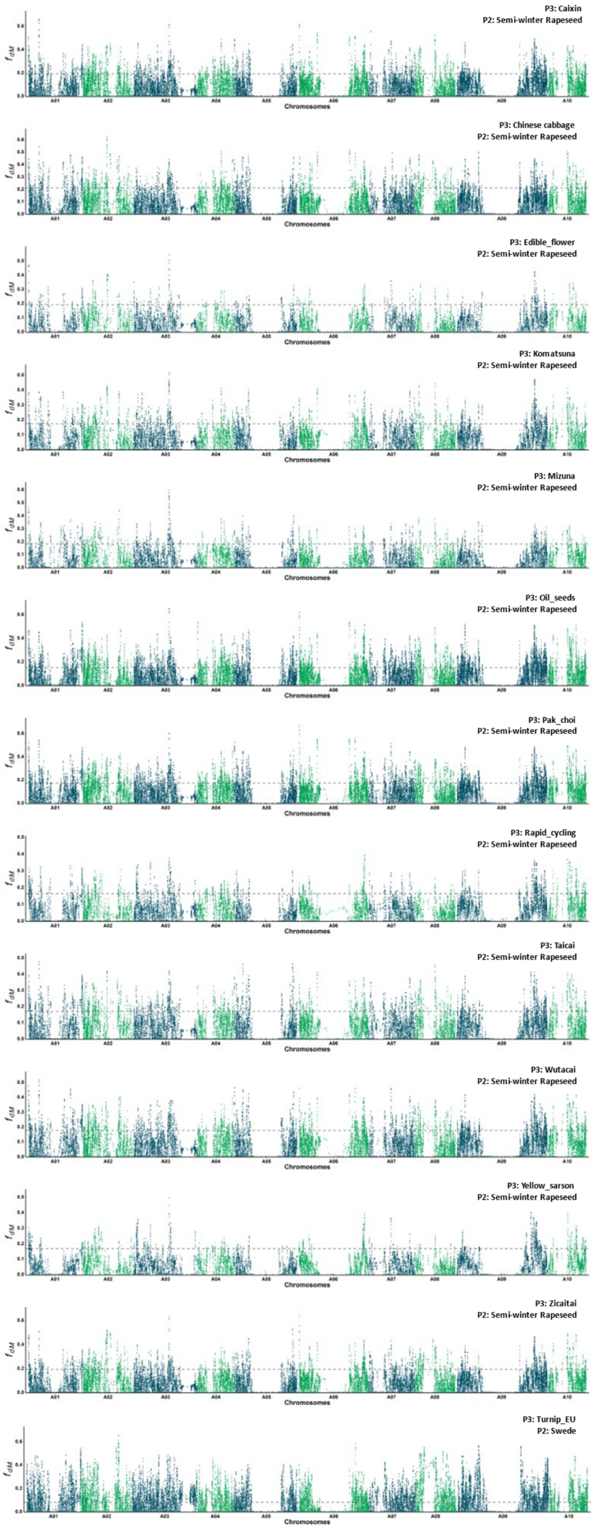

Supplementary Figure S18. Manhattan plot showing f_{dM} statistics across subgenome in A Lineage. The threshold line represents proportion of introgressed genome estimated by *f_4-ratio.*

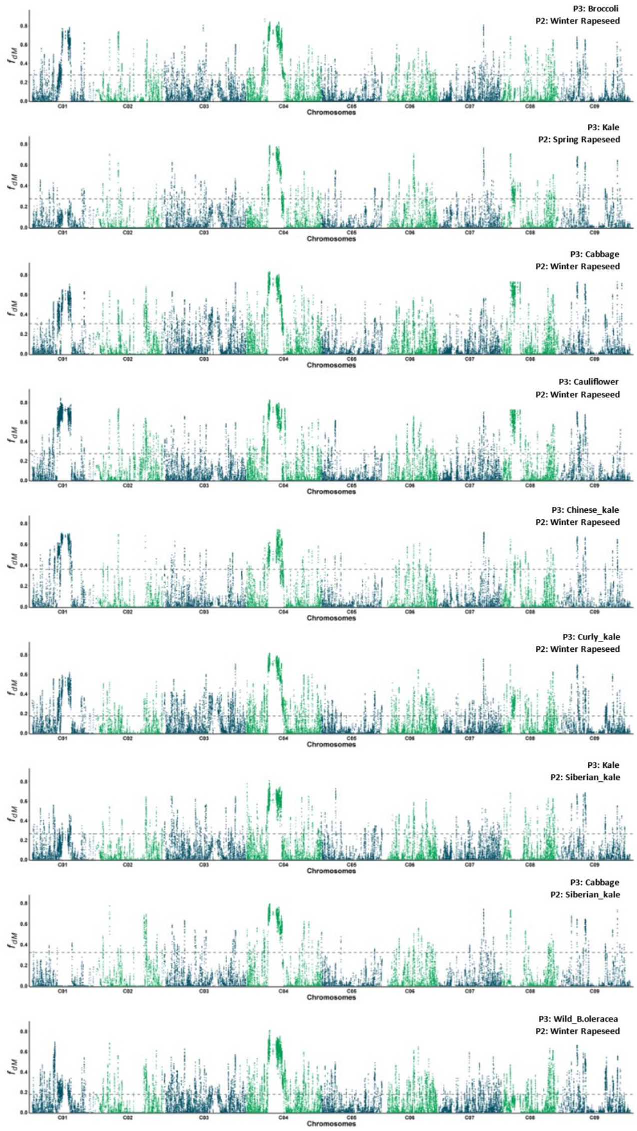

Supplementary Figure S19. Manhattan plot showing f_{dM} statistics across subgenome in C Lineage. The threshold line represents proportion of introgressed genome estimated by f_4*-ratio.*

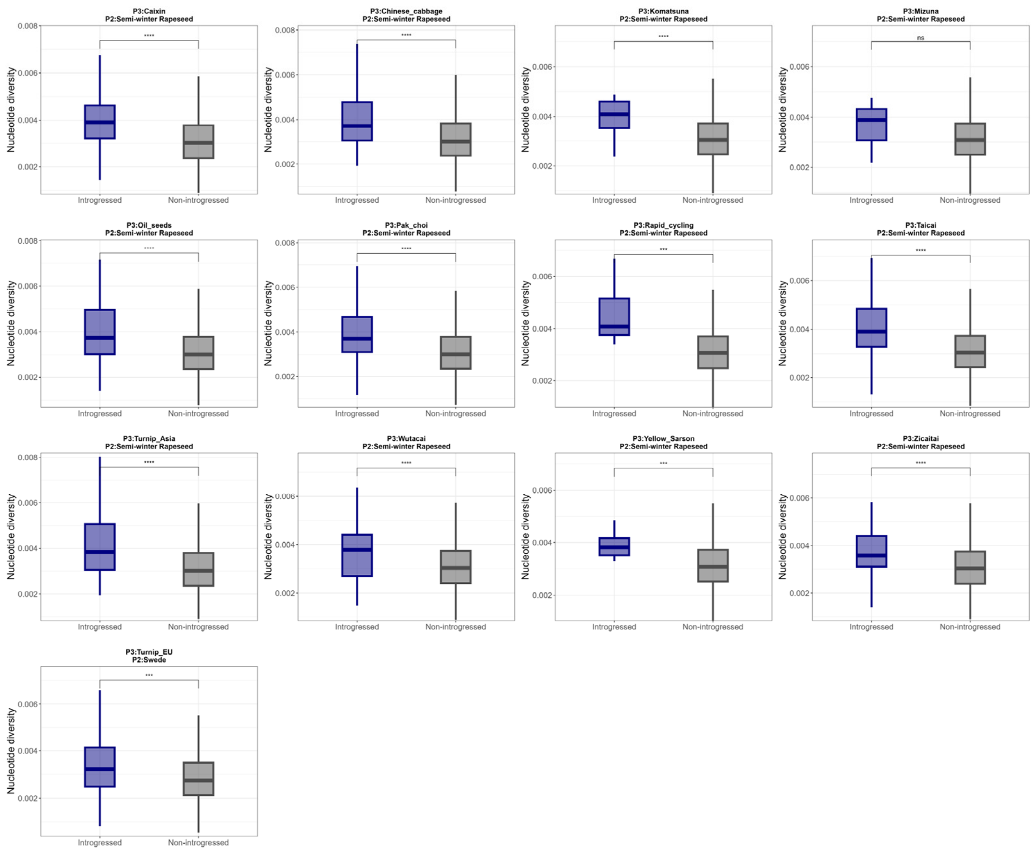

Supplementary Figure S20. Comparison of nucleotide diversity between putative introgressed and non-introgressed regions in A lineage. Mann-whitney tests were used to assess significance between introgressed and non-introgressed regions with asterisks indicating significance level. $^{***}P < 0.001$, $^{**}P < 0.01$, $^{*}P < 0.05$, $^{ns}P > 0.05$.

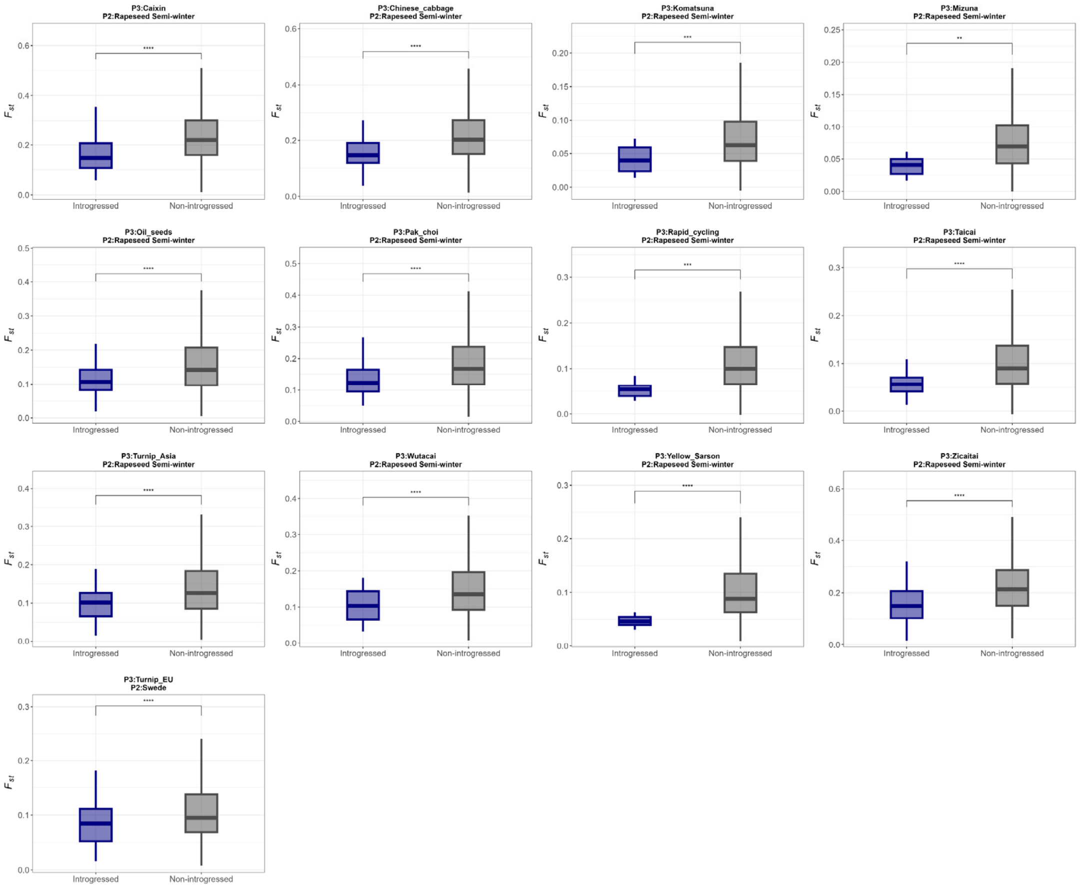

Supplementary Figure S21. Comparison of genetic difference (*F_{st}*) of donor and receptor between putative introgressed and non-introgressed regions in A lineage. Mann-whitney tests were used to assess significance between introgressed and non-introgressed regions with asterisks indicating significance level. $^{***}P < 0.001$, $^{**}P < 0.01$, $^{*}P < 0.05$, $^{ns}P > 0.05$.

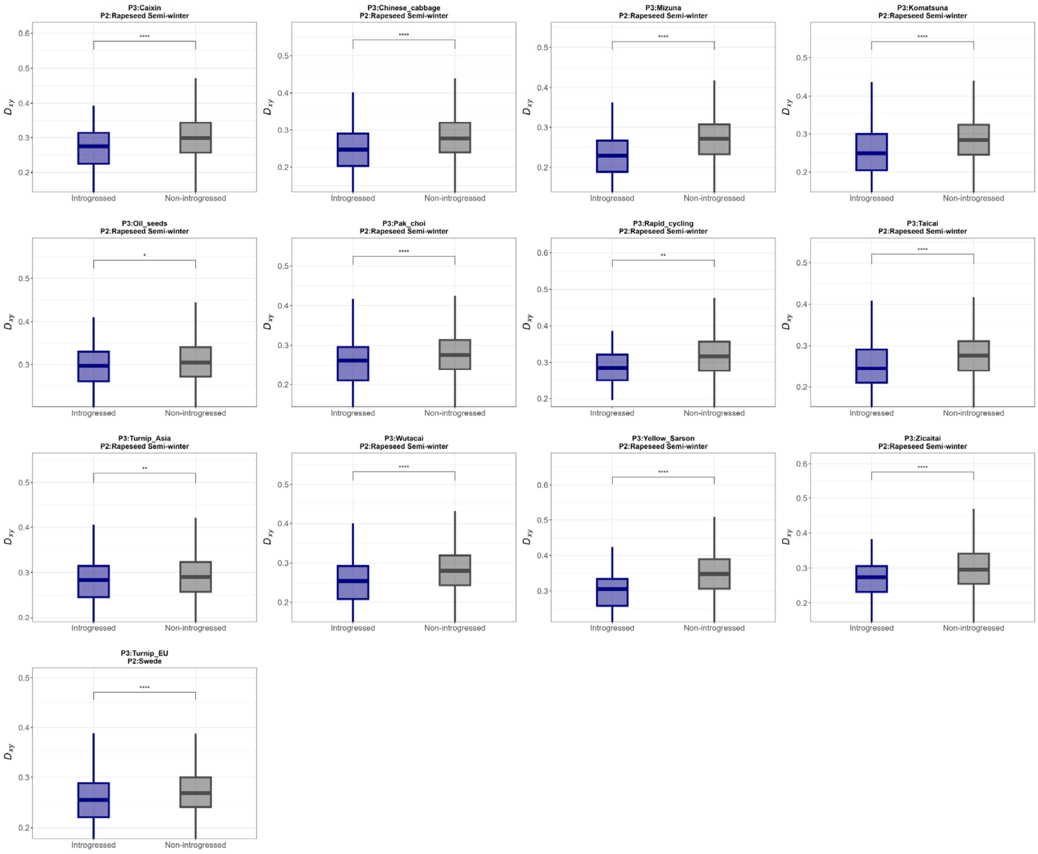

Supplementary Figure S22. Comparison of genetic difference (*Dxy*) of donor and receptor between putative introgressed and non-introgressed regions in A lineage. Mann-whitney tests were used to assess significance between introgressed and non-introgressed regions with asterisks indicating significance level. $^{***}P < 0.001$, $^{**}P < 0.01$, $^{*}P < 0.05$, $^{ns}P > 0.05$.

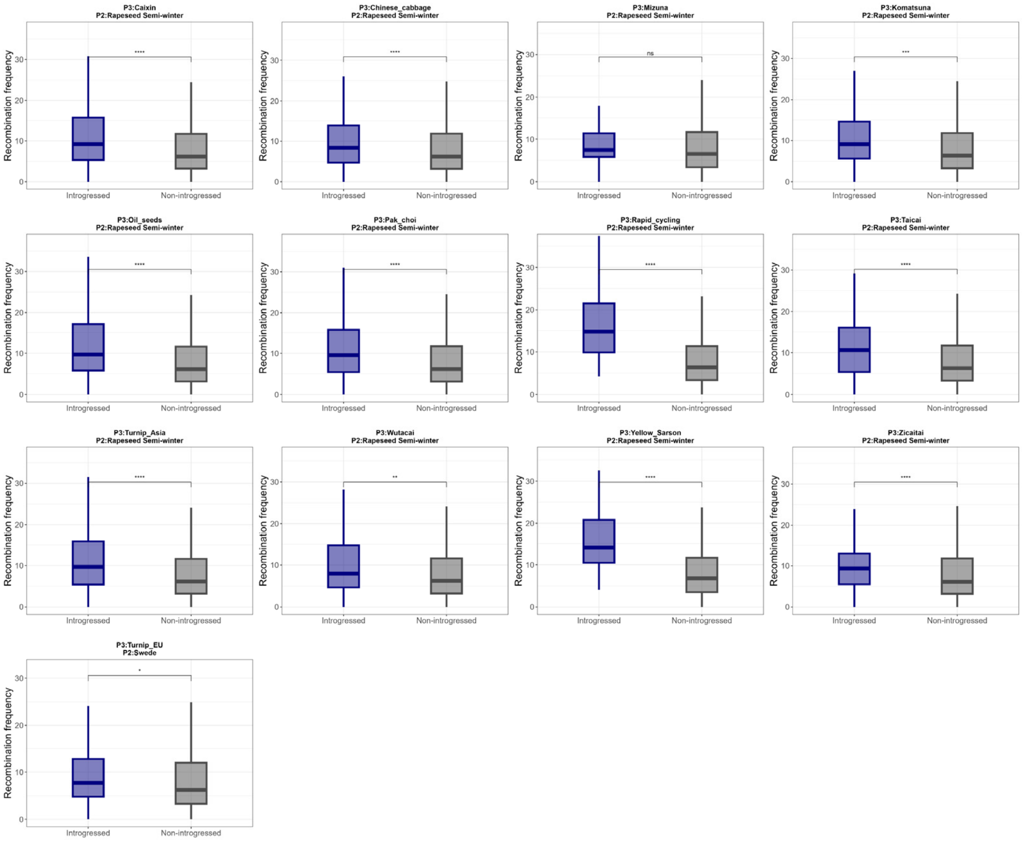

Supplementary Figure S23. Comparison of recombination frequency between putative introgressed and non-introgressed regions in A lineage. Mann-whitney tests were used to assess significance between introgressed and non-introgressed regions with asterisks indicating significance level. $^{***}P < 0.001$, $^{**}P < 0.01$, $^{*}P < 0.05$, $^{ns}P > 0.05$.

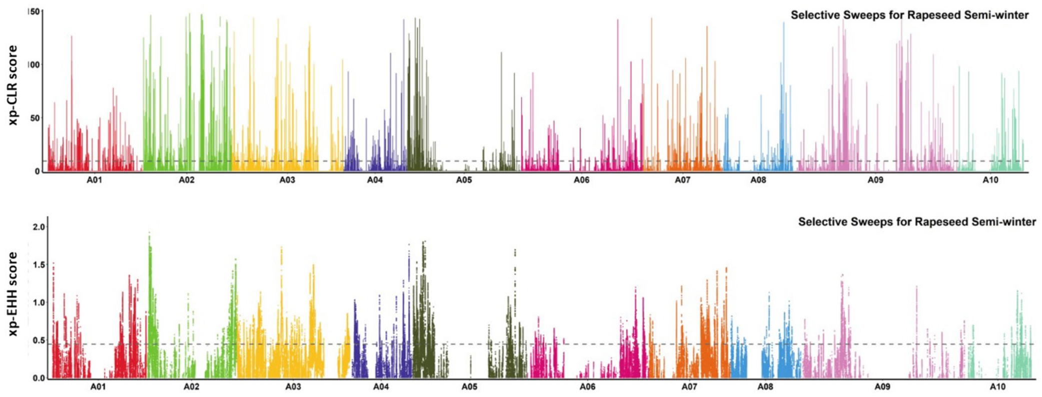

Supplementary Figure S24. Whole-genome scanning of the selection signals estimated by two haplotype-based statistics in semi-winter rapeseed compared with winter rapeseed. The horizontal grey lines refer to the top 5% threshold of selective sweeps**.**

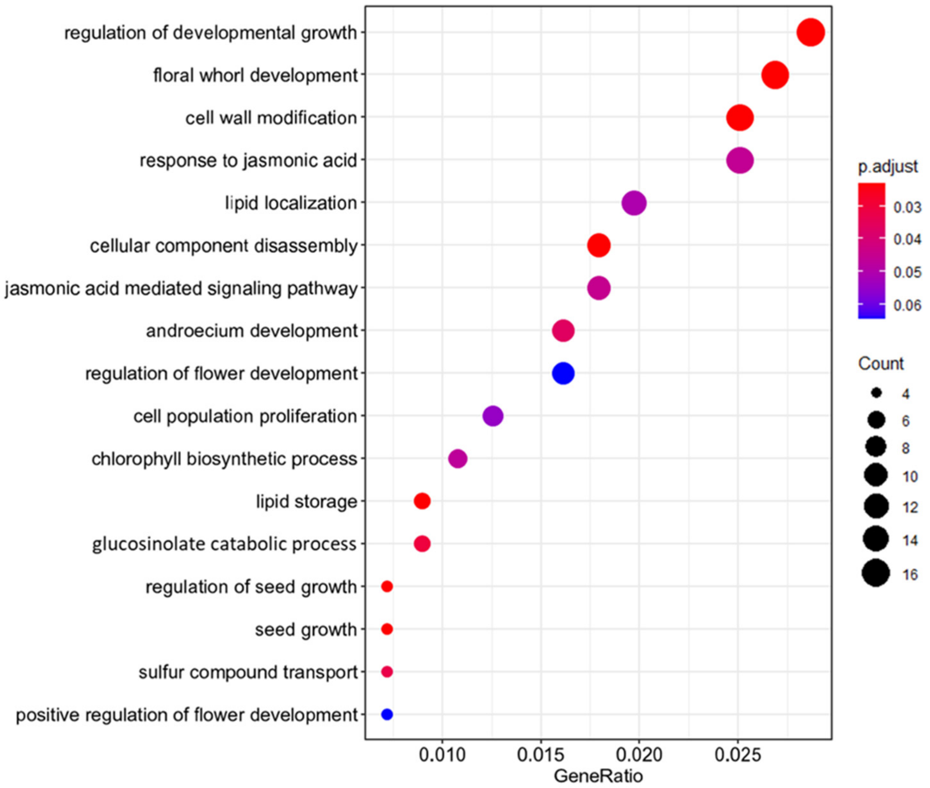

Supplementary Figure S25. GO enrichment analysis for selective introgressed genes during semi-winter rapeseed domestication.

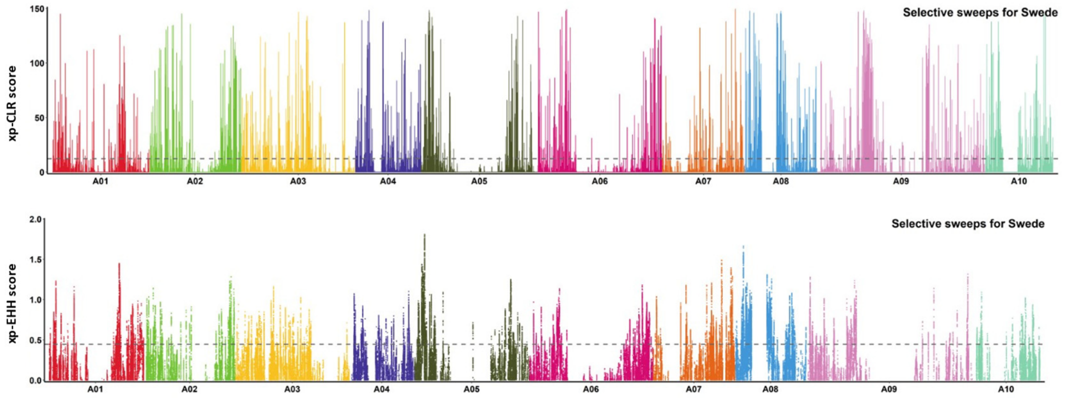

Supplementary Figure S26. Whole-genome scanning of the selection signals estimated by two haplotype-based statistics in swede compared with winter rapeseed. The horizontal grey lines refer to the top 5% threshold of selective sweeps**.**

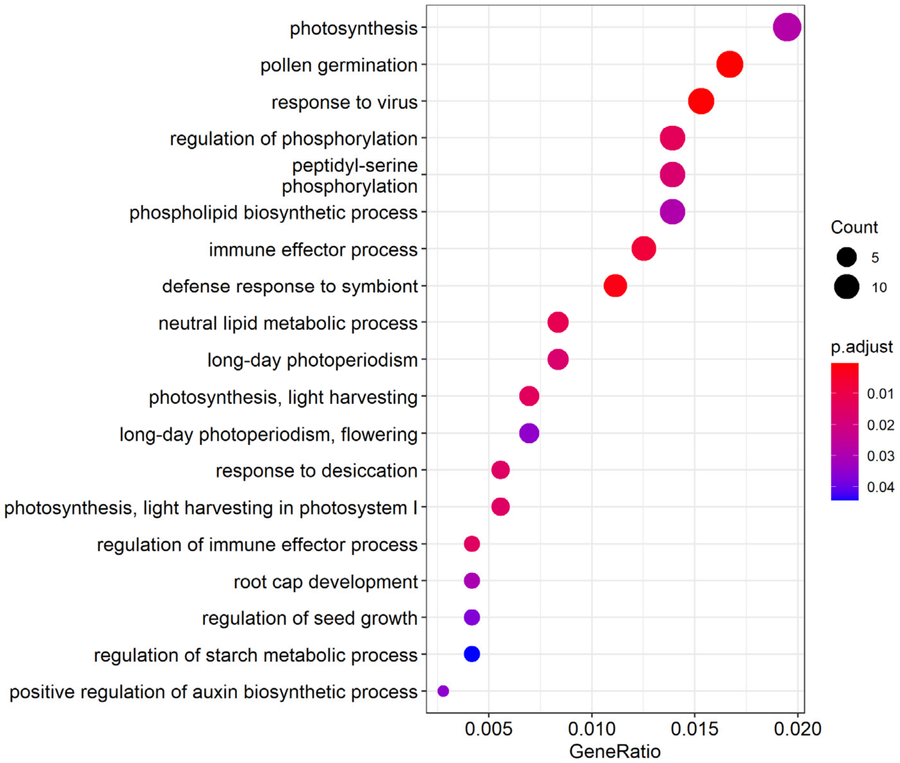

Supplementary Figure S27. GO enrichment analysis for selective introgressed genes during swede domestication.

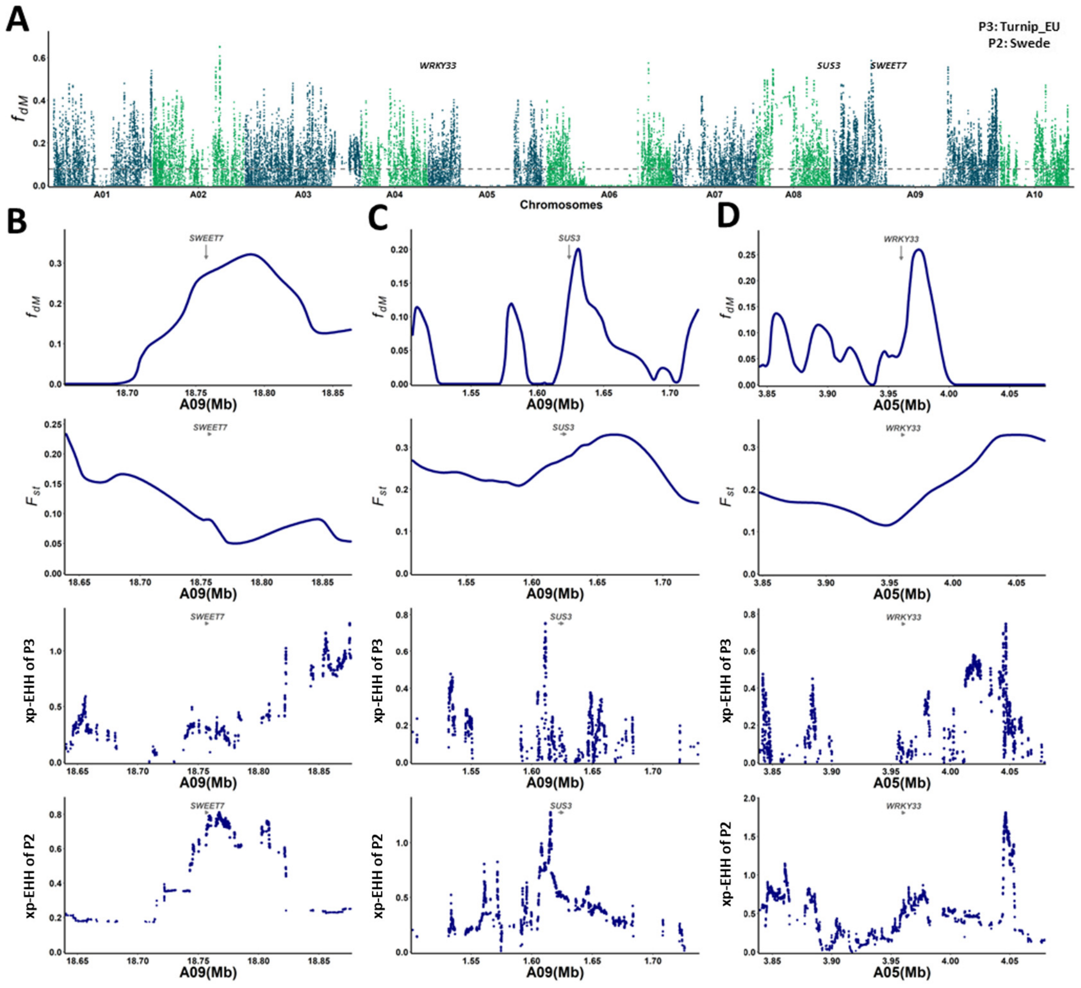

Supplementary Figure S28. Genomic characteristics of the putative introgressed regions in swede. (*A*) Manhattan plot showing the f_{dM} value across A subgenome. The dash line shows the cutoff value, calculated by highest x% of f_{dM} values, where x was determined by the corresponding f_4-ratio estimate. (*B, C, D*) Magnification of the representative adaptive introgression genes showed by f_{dM} and selective sweeps.

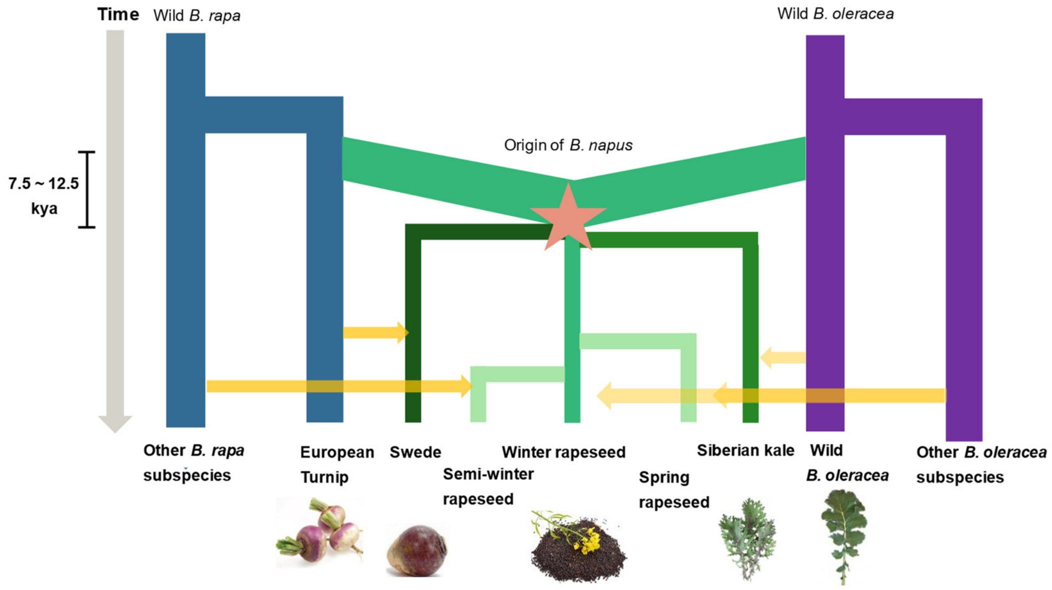

Supplementary Figure S29. Proposed model for phylogeny and demographic footprints of *B. napus*.
